# Lyophilized platelet derived extracellular vesicles promote hemostasis and attenuate intracranial hemorrhage following traumatic brain injury

**DOI:** 10.1186/s12967-026-08638-5

**Published:** 2026-07-20

**Authors:** Alpa Trivedi, Byron Miyazawa, Alexander T. Fields, Michael Matthews, Lindsay Vivona, Callie Keane, Daniel Potter, Huimin Geng, Haoqian Zhang, Hiroki Taenaka, Serena S. Kwek, Kimberly Herrera-Rodriguez, Brenda Nunez-Garcia, Erika Marques de Menezes, Mark Barry, Alison Nair, Ben Kuhn, Philip J. Norris, Lawrence Fong, Michael M. Fitzpatrick, Martin Schreiber, Lucy Z. Kornblith, Shibani Pati

**Affiliations:** 1 Department of Laboratory Medicine, University of California, San Francisco, 513 Parnassus Avenue, HSE 715, San Francisco, CA 94143 USA; 2 Department of Surgery, University of California, San Francisco, San Francisco, CA USA; 3 Cellphire Therapeutics, Inc, Rockville, MD USA; 4 Department of Anesthesia, Cardiovascular Research Institute, University of California, San Francisco, CA USA; 5 Division of Hematology/Oncology, Department of Medicine, University of California, San Francisco, San Francisco, CA 94143 USA; 6 Vitalant Research Institute, San Francisco, CA 94118 USA; 7 Department of Pediatrics, University of California, San Francisco, San Francisco, CA USA; 8 Department of Medicine, University of California, San Francisco, CA USA; 9 Fred Hutchinson Cancer Center, Seattle, WA USA; 10 Department of Surgery, Uniformed Services University, Bethesda, MD USA; 11 Present Address: Department of Anesthesiology and Intensive Care Medicine, Osaka University Graduate School of Medicine, Suita, Japan

**Keywords:** Traumatic brain injury, Exosomes, Blood brain barrier, Thrombin, Light transmission aggregometry, GPIIb/IIIa, Dysfunctional platelets

## Abstract

**Background:**

Hemorrhage is the leading cause of preventable death world-wide. Traumatic brain injury (TBI) is the primary driver of mortality and morbidity among individuals aged 1–44 years worldwide, with intracranial hemorrhage (ICH) being the major contributor to acute mortality post-TBI. Based on prior studies, we hypothesized that lyophilized platelet-derived extracellular vesicles (LPEVs) would reduce ICH and preserve blood-brain barrier (BBB) integrity after TBI.

**Methods:**

LPEVs were characterized by flow cytometry, scanning electron microscopy, and Nanosight. LPEVs were evaluated in vitro in human brain endothelial cell monolayers for barrier integrity. Utilizing a murine TBI model of controlled cortical impact, LPEVs were transfused 40 min after injury. The degree of BBB permeability was quantitated by 10kD infrared-tagged dye that leakage into the brain. ICH was quantitated by RBCs using Ter-119 antibody. Murine tail snip and cremaster muscle vascular injury models and thromboelastography analysis of healthy donor or trauma patient blood were used to assess hemostatic ability of LPEVs. Proteomic content was analyzed by mass spectrometry and mRNA and miRNA content by next-generation sequencing. Light transmission aggregometry using fresh apheresis platelets or generated dysfunctional platelets supported the mechanistic pathway assessment.

**Results:**

LPEVs were predominantly in the exosome range, expressed platelet and microvesicle markers and generated thrombin. In vitro, LPEVs attenuated thrombin-induced paracellular permeability and reduced intercellular gaps in human brain microvascular endothelial cells. In vivo, LPEVs exhibited potent hemostatic activity in a murine tail-transection and cremaster vascular injury models. Following experimental TBI in mice, acute administration of LPEVs significantly reduced ICH without impacting BBB permeability. Multi-omics analyses revealed that LPEVs retained platelet-derived hemostatic cargo, but lacked the vasculoprotective factors, typically present in intact platelets. LPEVs accelerated clot formation in blood from healthy donors, trauma patients, and dysfunctional platelet preparations. Mechanistically, LPEVs promote hemostasis in part through GPIIb/IIIa-dependent platelet aggregation.

**Conclusions:**

These data demonstrate the therapeutic and hemostatic efficacy of LPEVs in reducing ICH following TBI without affecting BBB permeability or neuroinflammation. The multi-omics and GPIIB/IIIA driven data can potentially explain the decoupled effects of LPEVs on hemostasis and vascular permeability in our experimental TBI model.

**Supplementary Information:**

The online version contains supplementary material available at https://doi.org/10.1186/s12967-026-08638-5.

## Introduction

Hemorrhage is the leading cause of preventable death world-wide [1–3]. Traumatic brain injury (TBI) is one of the most common causes of morbidity and mortality across both young and older populations and intracranial hemorrhage (ICH) is responsible for a large number of early TBI related deaths [4–7].

The initial impact in TBI can result in damage to the skull, followed by the development of ICH, and thrombus formation, leading to coagulopathy in some patients [8–13]. Approximately 20–30% of patients with isolated TBI present with coagulopathy and low platelets counts on admission [14], which are strong predictors of poor outcomes and increased risk of mortality [7, 15–17]. A substantial proportion of patients with TBI sustain concomitant injuries that result in hemorrhage and hemorrhagic shock, exacerbating cerebral injury and increasing mortality [15, 18]. Platelets are known key mediators of hemostasis and the platelet extracellular vesicles (PEVs) they produce have also been reported to be hemostatic [19, 20]. Platelet transfusion has been associated with improved survival in bleeding and coagulopathic patients [21, 22]. However, its clinical use is limited by a short shelf-life, stringent storage requirements, storage lesions, and limited availability or Blood Deserts world-wide [23–25]. Current blood banking protocols recommend that platelets be stored at room temperature with agitation for five to seven days or under cold storage for up to 14 days [26].

Platelet-derived extracellular vesicles (PEVs) offer a promising alternative. EVs are classified by size: large microvesicles (100–1000 nm) and small microvesicles (30–200 nm). PEVs constitute the majority of EVs in circulation and are key mediators of intercellular communication [27]. They carry a subset of platelet-derived RNA, proteins, and mitochondria, and their heterogeneity is determined by the activation stimulus that induces their release. PEVs are enriched in growth factors, cytokines, chemokines, anti-inflammatory molecules, and microRNAs [28]. In our past studies, we have shown that small microvesicle sized PEVs mitigate thrombin-induced disruption of tight and adherens junctions in endothelial cells, and support hemostasis in bleeding and vascular injury models [19, 20]. While PEVs may provide a hemostatic alternative to platelets when platelets are limited, fresh PEVs typically require storage at -20 °C for extended periods of time. In this paper we sought to investigate the potential of lyophilized PEV (LPEVs) that can be stored at room temperature without refrigeration, offering a logistically advantageous solution for long-term storage, availability and utilization in remote or austere environments. Here we elucidate the hemostatic properties of LPEVs in assays of hemostatic function both in vitro and in vivo utilizing murine models of hemorrhage.

In this study, LPEVs are isolated and dried using our methodology and displayed the size range of small microvesicles. LPEVs expressed platelet-specific and exosomal markers. Functionaly, LPEVs retained key platelet-derived activities, including the ability to support platelet aggregation, generate thrombin and significantly attenuate thrombin-induced paracellular permeability in human brain microvascular endothelial cells (BECs). These findings are consistent with our previous studies with fresh PEVs, where fresh PEVs promoted thrombin generation, increased clot strength, reduced abdominal blood loss following liver injury in rodents, and prevented the development of hemorrhagic shock in rodent models of trauma and hemorrhage [19]. Extending these observations, we demonstrate here that LPEVs are hemostatically active in vivo significantly reducing bleeding and promoting clot formation in both murine tail-transection and cremaster muscle vascular injury models respectively. Importantly, when administered intravenously following TBI, LPEVs reduced ICH. However, unlike certain platelet-derived biologics, LPEVs did not attenuate BBB permeability, astrocyte activation, or neuroinflammation [29]. Consistent with their hemostatic activity, LPEVs, similar to PEVs, enhanced clot formation in thromboelastography assays when added to whole blood obtained from both healthy donors and trauma patients. Omics-based analyses revealed that LPEVs retain key hemostatic proteins while reducing or eliminating several vasculoprotective factors. These findings could explain our noted difference between a platelet vs. LPEV effect. Mechanistically, we identified one such retained protein, GPIIb/IIIa, as a critical mediator of LPEV-induced platelet aggregation and clot formation. In contrast, GPIIb/IIIa did not appear to contribute to vascular stabilization, suggesting that the hemostatic and vasculoprotective effects of platelet-derived vesicles are potentially mediated through distinct molecular pathways. This study is the first to demonstrate the therapeutic and hemostatic efficacy of lyophilized platelet extracellular vesicles in reducing intracranial hemorrhage following traumatic brain injury. Furthermore, it is the first study to identify GPIIb/IIIa as a key mediator of the procoagulant activity of LPEVs, independent of vascular effects.

## Methods

### Preparation of lyophilized platelet derived extracellular vesicles (LPEV)

Platelet-derived extracellular vesicles (PEVs) and lyophilized PEVs (LPEVs) were acquired from Cellphire Therapeutics, Inc. (Rockville, MD) who developed a collection and processing method to manufacture PEVs per MISEV2018 guidelines [30]. Isolation of PEVs was performed as described before [31] and explained in detail in the supplemental section. Each lot was reconstituted in 1 ml of ultrapure sterile water and used within one hour of reconstitution.

### Characterization of LPEVs

#### Flow cytometry and nanoparticle tracking analysis (NTA)

LPEVs were characterized for the expression of platelet and EV related proteins by flow cytometry as described previously [20] and described in detail in the supplemental section.

#### Thrombin generation assay

Thrombin levels in LPEVs were measured as described previously [20] and described in detail in the supplemental section.

#### Scanning electron microscopy (SEM)

LPEVs were visualized by SEM (NanoCraft, Santa Clara, CA). LPEVs were seeded onto fibronectin-coated coverslips (Neuvitro Corp, Vancouver, WA) and allowed to attach at 22℃ for 30 min. Coverslips were washed with PBS, fixed with 4% glutaraldehyde, washed with phosphate buffered saline (PBS), and fixed with 2% osmium tetroxide (OsO4). Following this, coverslips were freeze-dried, and gold sputtered. These coverslips were imaged using an SEM (TESCAN Vega-3, Czech Republic) at 10,000x magnification.

### Platelet and LPEV content analysis

#### Proteomic and transcriptomic analysis of Platelets and LPEVs

Sample preparation and methodology for liquid chromatography-tandem mass spectrometry (LC-MS/MS) for proteomic analysis and library generation and sequencing for transcriptomic content analysis as described previously [29] and are described in detail in the supplemental section. Statistical analyses used are described in the section below.

#### Quantitation of endothelial barrier permeability in monolayer cell culture

Human brain microvascular endothelial cells (BECs) (Angio-Proteomie, Boston, MA) were expanded and maintained using Endothelial Growth Medium (Angio-Proteomie). The integrity of BEC monolayers was measured using an electric cell-substrate impedance sensing system (ECIS 1600, Applied BioPhysics, Troy, NY) as described previously and described in detail in the supplemental Sects. [20, 29, 32, 33].

#### Quantitation of gap formation in BEC monolayers

BECs were grown to confluence on 96-well Image-Lock plates (Sartorius, Göttingen, Germany). LPEVs (10^11^ particles/ml) were added to the BEC monolayers for 30 min, followed by a 2U/mL thrombin challenge for 5 min at 37 °C. Images of the BEC monolayers were captured every 20 min for 3 h using the Incucyte SX3 (Sartorius). Confluence measurements of the BEC monolayers were analyzed by the Incucyte SX3 software.

#### Animal protocols

All the animal studies were performed with approval of the Institutional Animal Care and Use Committee at UCSF (Protocol approval number: AN199625-00B). The experiments were conducted in compliance with the National Institutes of Health guidelines on the use of laboratory animals and the Department of Defense Animal Care and Use Review Office. All animals were housed in a room with access to food and water *ad libitum*, controlled temperature and 12:12-hour light-dark cycles. Mice in each of the individual assays were randomized to injury and treatment. The surgeon was blinded to the treatments and all histological evaluations, and blood loss measurements were performed blinded to treatment and injury.

#### Intravital microscopy of mouse cremaster vasculature injury model

Adult male C57BL6 mice (8–12-week-old) (Jackson Laboratories, Bar Harbor, ME) were used for this study. Surgery and measurements were performed as described previously [29, 33] and in detail in the supplemental section.

#### Tail snip assay

The tail snip bleeding model was performed in 8- to 10-week-old male mice (Jackson Laboratories) as described previously [20] and described in detail in the supplemental section.

#### Traumatic brain injury (TBI) model

The controlled cortical impact (CCI) injury was performed on 10–12 week old C57Bl/6J male mice (Jackson Laboratories) to measure BBB permeability and tissue histopathology as described previously [29, 34–36] and in detail in the supplemental section.

#### LPEV functional assays in human whole blood

All human participant study procedures were approved by the University of California, San Francisco Institutional Review Board and the Department of Defense Human Research Protection Office. Under an IRB-approved waiver of consent (IRB 19-29291), all whole-blood LPEV functional assays were performed in real time immediately after blood collection in the Emergency Department (ED). For injured patients, blood samples were collected under an IRB-approved waiver of informed consent because samples were obtained during emergency trauma resuscitation, the research posed minimal risk, and participants were frequently unable to provide consent at the time of enrollment. Injured patients or their legally authorized representatives were subsequently informed of study participation and given the opportunity to consent to continued clinical data collection when feasible. Written informed consent was obtained from all healthy volunteer control participants prior to participation.

Samples from trauma patients were obtained upon arrival to the ED at the time of intravenous access placement, and assays were initiated without delay. Healthy donor samples underwent the same immediate processing workflow, ensuring minimal delay from collection to assay was a critical component of the study design. Whole blood samples were mixed with matched volume saline, PEV, or LPEV (both to final concentration of 10^9^-10^11^ particles/mL) immediately prior to performing thromboelastography (TEG, Haemonetics, Braintree, MA). TEG was performed on treated blood samples with kaolin activation.

#### Light transmission aggregometry (LTA) in apheresis platelets

Day 1 leukoreduced platelets (Plts) (Vitalant, Denver, CO) from 3 donors were pooled, centrifuged at 800 g for eight minutes and resuspended in a fixed volume of PBS. Platelets were prepared and treated with agonist to measure aggregation. This method was also used to dissect the role of GPIIb/IIIa pathway in LPEV induced aggregation in apheresis and dysfunctional platelets, as described in the supplemental methods section.

#### Statistical analyses

All statistical analysis for in vitro and in vivo experiments was performed by UCSF staff (AT, BM, and CK) that were not associated with Cellphire. Measures of trans endothelial electrical resistance (TEER), and gap formation were analyzed by One-way ANOVA, followed by Tukey’s multiple comparisons tests. Light aggregometry and in vivo BBB permeability measures were analyzed by repeated measures (RM) two-way ANOVA and Tukey’s post-hoc tests. Two group comparisons were made by unpaired two-tailed t-test. All statistical analyses were performed using GraphPad Prism 10 software, (GraphPad Software, La Jolla, CA). Differences between groups were considered significant with *p* ≤ 0.05.

Statistical analysis for the patient sample analysis - TEG studies were performed by UCSF staff (AT, AF, and CK) that were not associated with Cellphire. For comparison of TEG results between groups, patients were stratified by their injury status (injured or healthy), presence of shock (shock index≥1), and presence of a traumatic brain injury (based on chart review). Patients were excluded if they were found to be pregnant, incarcerated, minors, or on anticoagulant or antiplatelet medication. TEG data were excluded as outliers if they were less than 1.5 times the interquartile range (IQR) below Q1 or greater than 1.5 times the IQR above Q3. Statistical significance was then assessed by mixed model analyses of comparison groups. Mixed-effects modeling was selected based on GraphPad Prism’s recommended approach for our data structure. The model allowed us to account for both fixed effects (e.g., treatment condition) and random effects (e.g., individual patient variation). Differences between groups were considered significant with *p* ≤ 0.05.

These statistical analyses were performed by UCSF staff (HG) who was not associated with Cellphire. For transcriptomic and RNA-seq analysis, sequence alignment was performed using Bowtie2 [37] local mode alignment with default parameters. The FASTQ files were mapped to the human reference genome using the Ensembl GRCh38 cDNA and non-coding RNA combined reference. Normalized read counts and differential expression were calculated for each transcript by DESeq2 [38]. Differentially expressed genes between two groups were presented in heatmap using R software version 4.0.2 [39]. Differences between groups were considered significant with *p* ≤ 0.05. For Pathway Analysis: KEGG, BioCarta and GO pathway enrichment analyses were subsequently conducted on the differentially expressed genes using the DAVID software [40]. Pie distribution plots of the significant pathways (FDR < 0.05) were generated using the Plotly package in R software [39].

## Results

### LPEVs display functional hemostatic potential

**Fig. 1:**
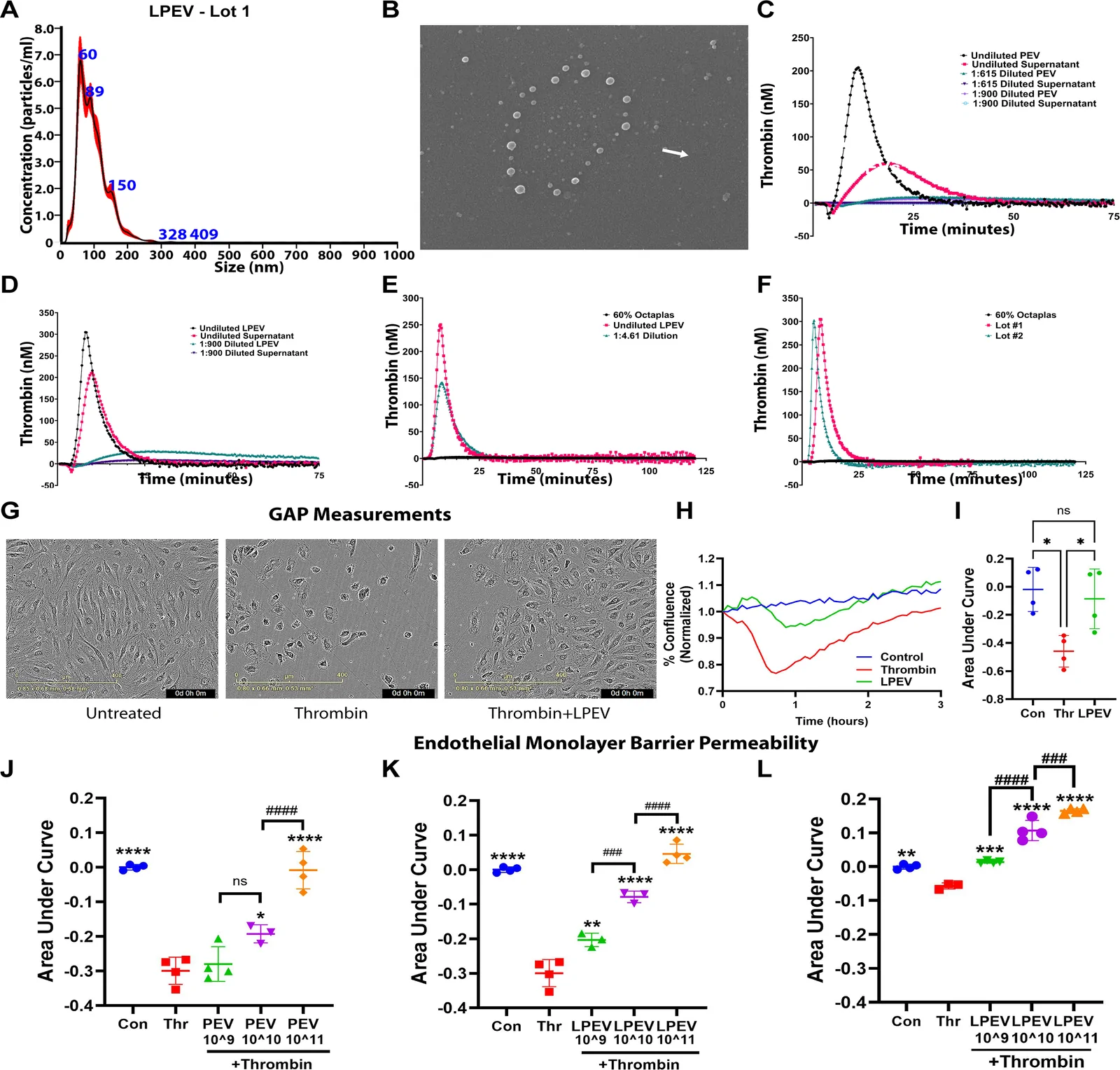
In vitro characterization and functional assessment of LPEVs. (**A**) A majority of extracellular vesicles were in the size range of small microvesicles (approximately 100 nm). (**B**) Scanning electron microscopy (SEM) analysis confirmed the size of the bulk of the particles as around 100 nm. (**C**) Functional analysis of platelet-derived EVs (PEVs) demonstrate the ability to generate thrombin as detected by thrombin generation assay (TGA) in a dose dependent effect that is defined by the presence of PEVs. (**D**) Functional analysis of LPEVs demonstrate their ability to generate thrombin in a dose dependent manner and that is defined by the presence of LPEVs. (**E**) LPEVs had the capacity to generate thrombin when added to 60% plasma (Octaplas), to supply the coagulation factors. Furthermore, Octaplas is detergent treated, and hence cannot on its own contribute to thrombin generation. EVs are the only source of phospholipids in the TGA assay, so all the thrombin generation activity from the TGA testing is caused by the EV product. Octaplas is not added to the EV product as part of the manufacturing process. Octaplas was not used in any of the other testing besides TGA. (**F**) Two different LPEV lots generated thrombin. (**G**) Representative still images of human BECs from each of the groups for gap measurements. (**H**) Graphical representation of gap area. (**I**) One-way ANOVA with Tukey’s post-hoc analysis, *p* = 0.0095, F [2, 9] = 3.701, *p = 0.0114 (con vs. Thr (Thrombin)), *p = 0.0273 (Thr vs. LPEV). (**J**) PEVs mitigate thrombin induced permeability in BECs in a dose dependent fashion. One-way ANOVA with Tukey’s post-hoc analysis, *p* < 0.0001, F [4, 14] = 1.098, * vs. thrombin (Thr), # between group comparisons, *p = 0.0254, ****p < 0.0001, ####*p* < 0.0001. (**K**-**L**) Two separate lots of LPEVs mitigate thrombin induced permeability in BECs in a dose dependent fashion. (**K**) One-way ANOVA with Tukey’s post-hoc analysis, *p* < 0.0001, F [4, 13] = 1.004, * vs. thrombin (Thr), # between group comparisons, **p = 0.0021, ****p < 0.0001, ###*p* = 0.0007, ####*p* < 0.0001. (**L**) One-way ANOVA with Tukey’s post-hoc analysis, *p* < 0.0001, F [4, 14] = 1.406, * vs. thrombin (Thr), # between group comparisons, **p = 0.0016, ***p = 0.0002, ****p < 0.0001, ###*p* = 0.0004, ####*p* < 0.0001. *N* = 3–4 wells per condition/experiment, with 2–3 experimental repeats (**I**-**L**)

Although the goal of our studies is not to compare PEVs to LPEVs, we conducted some initial characterization and functional experiments, described in our previous studies, to establish equivalence [19, 20]. Utilizing our methodology of isolation, we find that LPEVs predominantly exhibit a small microvesicle or exosomal size profile, as determined by Nanosight, scanning electron microscopy (SEM), and flow cytometry (Fig. 1A and B and Supplemental Figs. 1,  2), confirming a mean particle size of approximately 100 nm. These vesicles expressed the platelet marker CD41, along with exosome markers CD9 and Annexin V (Supplemental Fig. 2). We find that PEVs generate thrombin in a dose dependent fashion, supporting the hemostatic potential. Furthermore, PEV depleted supernatants generate lower amounts of thrombin, confirming the contribution of PEVs in thrombin generation (Fig. 1C). Similar dose dependent thrombin generation was observed with LPEVs and effect decreased in tested supernatants (Fig. 1D and E) and across multiple LPEV lots (Fig. 1F). In these studies, one lot is made of a pool of 4 donors. Based on our previous studies, platelets and platelet derived products [20, 29, 32, 33, 41, 42] pooling eliminates donor to donor variability in function- a desired trait for a clinical product.

### LPEVs protect human brain endothelial cell barrier permeability in vitro

Similar to our previously reported findings with PEVs, LPEVs attenuated thrombin-induced disruption of BEC monolayer integrity, as evidenced by reduced gap formation (Fig. 1G-I, and Movies 1–3). PEVs (Fig. 1J) and LPEVs (Fig. 1K) inhibited thrombin-induced BEC permeability in a dose-dependent manner. This protective effect was consistent across multiple lots (Fig. 1L).

### LPEVs are hemostatic in a murine model of bleeding and vascular injury

**Fig. 2:**
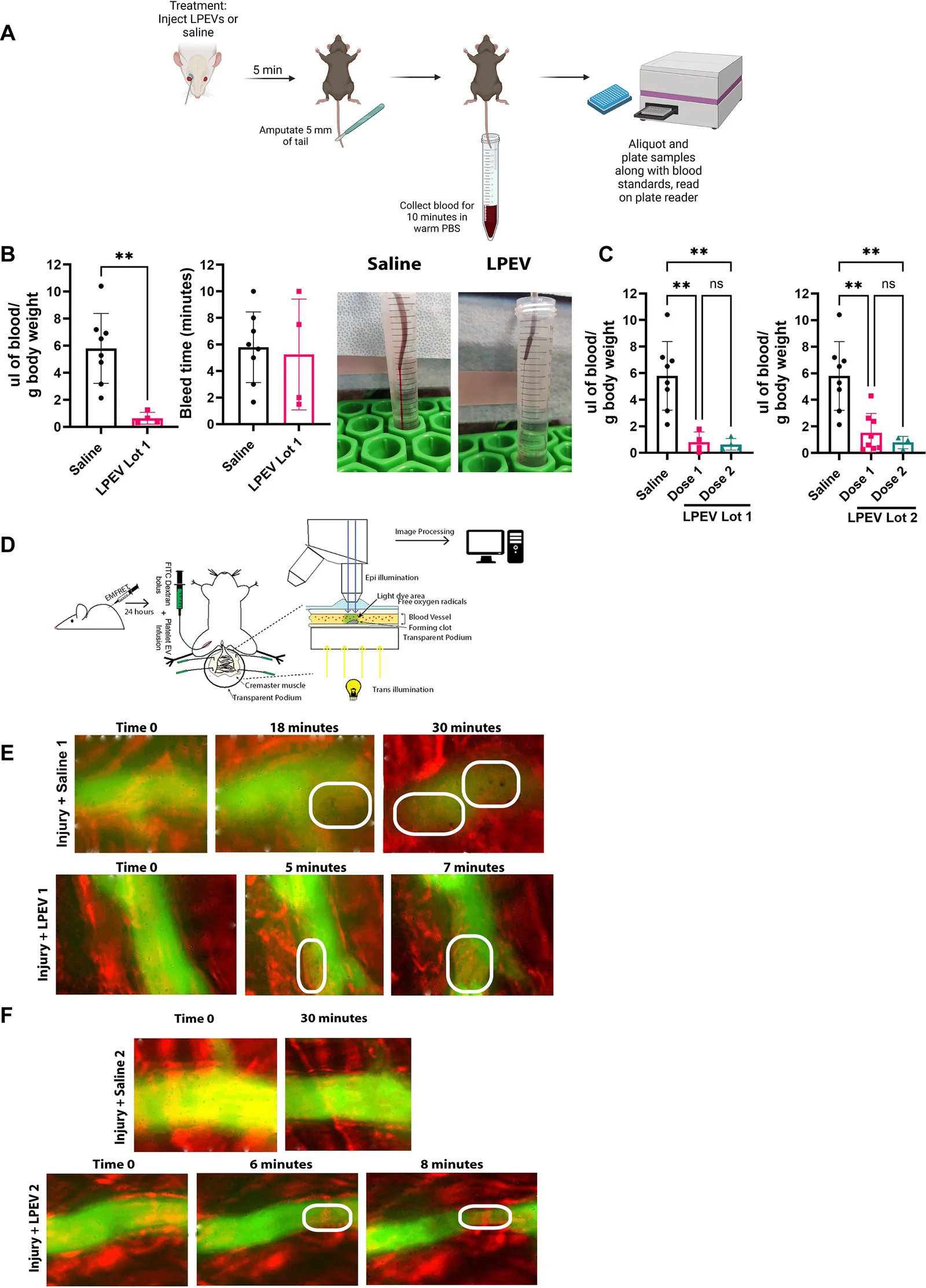
LPEVs reduce bleeding in a murine tail-snip model and induce clot formation in damaged blood vessels of cremaster muscle. (**A**) Experimental outline for tail snip assay.** (B)** LPEV (2 × 10^10^ particles/g body weight) treated mice had significant reduction in total blood loss corrected for body weight as compared to saline treated animals, unpaired two-tailed t-test, **p = 0.0031, F [3, 7] = 38.01, without affecting in bleeding time (unpaired two-tailed t-test, *p* > 0.05, *n* = 4 mice per group). Representative images from each group are shown on the right. **(C)** Two lots (different pooled donors) and multiple doses of both the lots showed that LPEV treated mice had significantly less total blood loss corrected for body weight as compared to saline treated mice. *Lot 1* was tested at dose 1: 4 × 10^8^ particles/g body weight and dose 2: 2 × 10^10^ particles/g body weight, One way ANOVA, *p* = 0.0006, F [2, 13] = 3.620, **p < 0.01. *Lot 2* was tested at dose 1: 4 × 10^8^ particles/g body weight and dose 2: 8 × 10^8^ particles/g body weight, One way ANOVA, *p* = 0.0006, F [2, 16] = 2.286, **p < 0.01, *n* = 4–8 mice per group. Multiple cohorts of animals were run. **(D)** Experimental outline showing that the endogenous platelets were depleted in the mice with GP1bα targeting antibody, 24 h prior to imaging. FITC dextran was injected via femoral vein along with treatment (LPEV or saline). Light induced injury was created in the blood vessel and time to clot formation and flow cessation was measured using Metamorph software. **(E)** Blood vessel in a saline treated animal takes 30 min to form a clot whereas LPEV treated animal forms clot in 5 to 7 min. **(F)** Another saline treated animal that shows absence of clotting ability even after 30 min of imaging. Whereas LPEV treated animal shows clot formation at 6 min and cessation of flow at 8 minutes post-injury. Three animals per group with multiple blood vessels imaged per animal. *[Experimental outline created with BioRender.com]*

Focusing on our LPEV isolates in vivo, utilized a tail snip murine bleeding model to evaluate the hemostatic efficacy of LPEVs (Fig. 2A). Treatment with LPEVs resulted in a significant reduction in blood loss compared to saline-treated mice, without altering the total bleeding time **(**Fig. 2B**)**. This protective effect was observed to be independent of both dose and lots (Fig. 2C). Supporting these findings is qualitative clot formation data in a cremaster model of vascular injury that reveals in LPEV-treated thrombocytopenic mice, time to clot and cessation of flow is decreased to approximately 8 min for LPEVs vs. 30 min for saline treated mice at the site of injury (Fig. 2D and F).

### LPEVs reduce TBI induced ICH without affecting BBB permeability and neuroinflammation

**Fig. 3:**
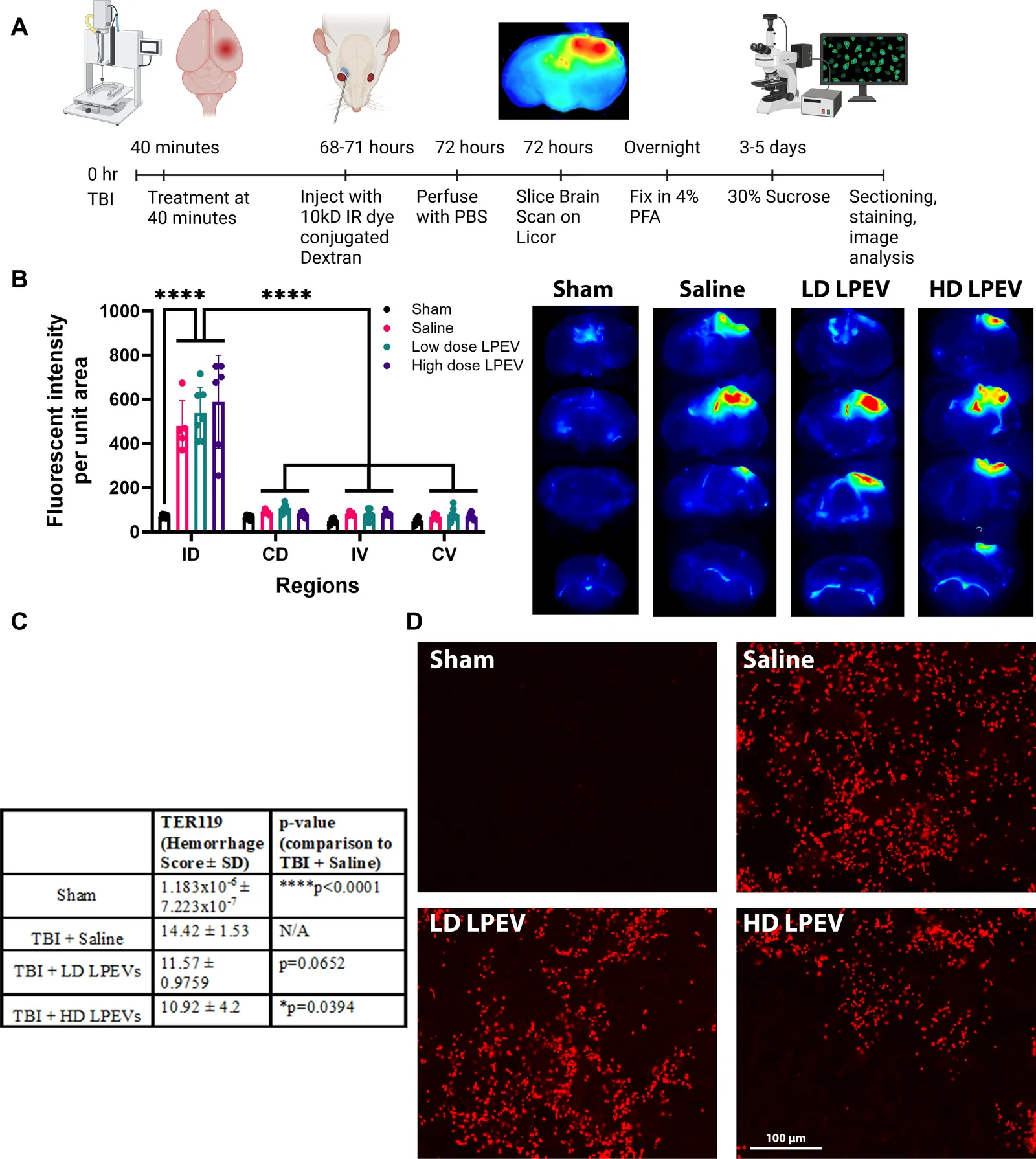
LPEVs decrease TBI induced hemorrhage in a dose dependent fashion, without affecting BBB permeability at 3 days post injury. (**A**) Experimental outline for evaluation in TBI. (**B**) There was an increased amount of dextran in the ipsilateral dorsal quadrant of the TBI group as compared to the other 3 quadrants within the group and as compared to ipsilateral quadrant of the sham group. RM two-way ANOVA, significant region x injury/treatment interaction, *p* < 0.0001, F [9, 60] = 18.19; significant effect of region, *p* < 0.0001, F [3, 60] = 169, and significant effect of injury/treatment, *p* < 0.0001, F [3, 20] = 18.53, columns represent mean ± SD, *n* = 5–7 per group. Representative scans from each group are presented on right. Both the LPEV treated groups were not different from each other or the saline treated group. ID: ipsilateral dorsal, CD: contralateral dorsal, IV: ipsilateral ventral, CV: contralateral ventral. (**C**) There is injury induced hemorrhage in the brain and high dose (HD) of LPEV treatment reduces hemorrhage as compared to saline treated group. One way ANOVA, *p* < 0.0001, F [3, 21] = 48.23, *n* = 6–7 per group, post-hoc comparison to saline group is shown in the table. (**D**) Representative images from each group. *[Experimental outline created with BioRender.com]*

Mice were randomized to injury and treatment - vehicle (saline), LPEVs at low dose (~ 3 × 10⁹ particles/g body weight), or high dose (~ 2 × 10¹⁰ particles/g body weight) - administered at 40 min post-injury, representing a pre-hospital administration in clinical setting. BBB permeability and ICH were assessed at 3 days post-injury (Fig. 3A). Increased BBB permeability observed in the ipsilateral dorsal quadrant of the injured mice was not significantly altered with LPEV treatment as compared to saline-treated mice (Fig. 3B). Notably, LPEV treatment at the higher dose reduced TBI-induced hemorrhage, with a strong trend toward reduction at the lower dose (Fig. 3C-D). Regarding neuroinflammatory endpoints, LPEV treatment did not reduce the infiltrating macrophage response, indicated by CD68 staining, at 3 days post-injury, as compared to saline controls **(**Supplemental Fig. 3A). Similarly, injury-induced activation of the resident immune cells (microglia - Iba1 expression), within the cortex and hippocampus, was unchanged between saline and LPEV-treated groups (Supplemental Figs. 3B– 3C). TBI-induced astrocyte activation - increased GFAP expression in the cortex, hippocampus, and white matter, was also not attenuated by LPEV treatment at either dose (Supplemental Fig. 4).

### Proteins related to coagulation are enriched whereas vasculoprotective proteins are depleted in LPEVs

**Fig. 4:**
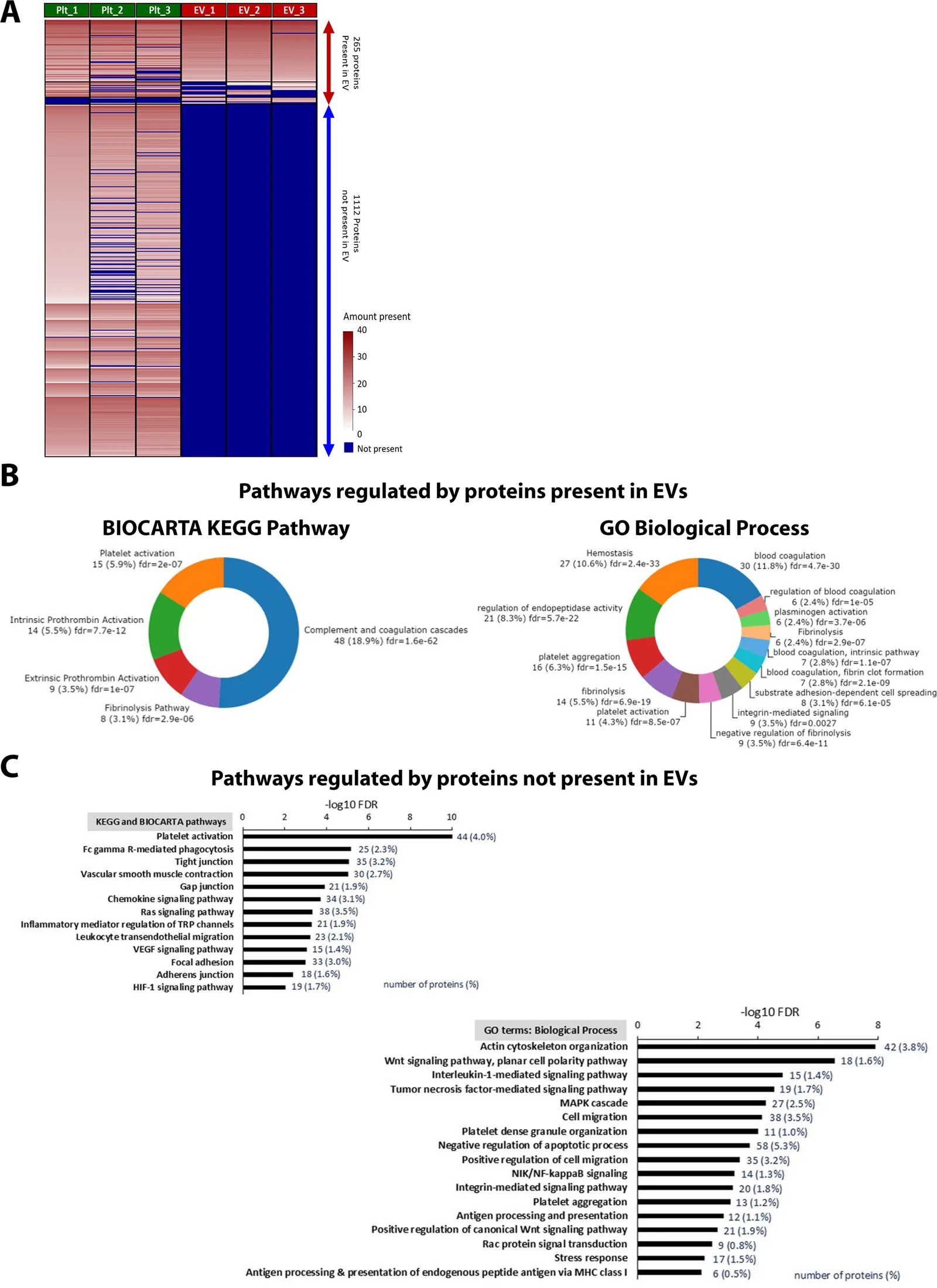
Proteomic evaluation of platelets and LPEV content highlights the differential functionality of LPEVs. (**A**) Heat maps of detected proteins show that while 1357 proteins were detected in the platelets, only 265 were present in the LPEVs (three lots tested). (**B**) Biocarta, KEGG and GO molecular pathway analysis are represented as donut plots, based on the proteins present in the LPEVs. Proteins that control the pathways related to platelet activation, both intrinsic as well as extrinsic, fibrinolysis, hemostasis, coagulation, platelet aggregation, clot formation and adhesion are present in the LPEVs as well as the platelets. (**C**) Biocarta, KEGG and GO molecular pathway analysis are represented, based on the proteins that are absent in the LPEVs. Y-axis represents the pathways that are affected, and x-axis is -log 10(FDR), number and % of proteins are highlighted next to each of the bar plots. Proteins that control the pathways related to inflammation, tight junction regulation, VEGF signaling, endothelial function, and wnt signaling are absent in the LPEVs but present in the platelets

To better understand the functional effects of the LPEVs, we conducted a proteomic analysis of their content and compared it to intact platelets (3 per group). Proteomic analysis of LPEVs and platelets identified over 1,300 proteins and peptides in platelet samples. Of these 1,300 proteins, only 265 were detected in LPEVs used in this study (Fig. 4A). Pathways regulated by proteins existing in LPEVs were found to be related to platelet activation, coagulation, intrinsic and extrinsic prothrombin activation pathways, platelet aggregation, and fibrinolysis, all of which are central to maintaining hemostasis (Fig. 4B). Some of the proteins that are present and conserved between platelets and LPEVs and involved in the coagulation cascade are Factor V, Factor X, Factor IX, Factor XII, Factor XIII, prothrombin, antithrombin-III, and plasminogen. In contrast, proteins present in platelets but absent in LPEVs were associated with functions including immunomodulation, leukocyte transendothelial migration, tight and adherens junction integrity, smooth muscle contraction, VEGF signaling, platelet structural maintenance and migration, WNT signaling, and TNFα signaling, pathways related to endothelial integrity and inflammation (Fig. 4C). Some of the proteins that are absent in LPEVs but present in the platelets and involved in vascular stability are PDGF, TIMP3, TIMP1, CD151, ESAM, ITGA5, ANGPT1, VWF, TGFB1, AND ADAM10. Proteins involved in leukocyte transendothelial migration and VEGF signaling that were absent in LPEVs are ITGB1, CDC42, JAM3, ROCK1 and 2, CLDN5, PECAM1, and ESAM.

### mRNAs and miRNAs related to hemostasis are enriched in LPEVs

**Fig. 5:**
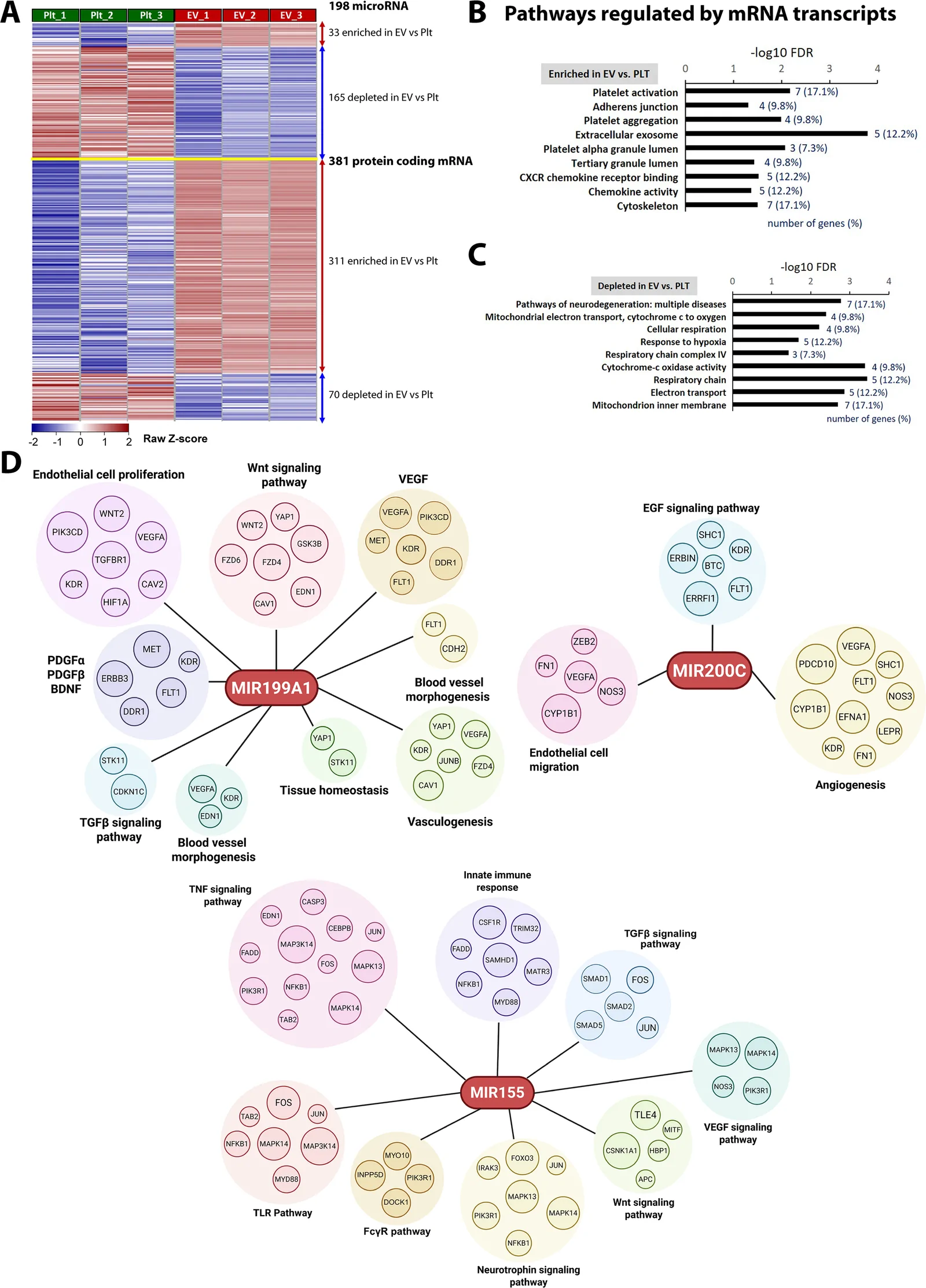
Transcriptomic evaluation of Plts and LPEV content. (**A**) Heat maps of mRNAs show that 381 protein coding RNAs and 198 miRNAs were detected. Of these 311 mRNAs were enriched and 70 depleted in the LPEVs. For miRNA, 33 were enriched and 165 were depleted in LPEVs as compared to platelets (three lots tested). (**B**) Biocarta, KEGG and GO molecular pathway analysis are represented, based on the mRNAs that are enriched or depleted in the LPEVs. Y-axis represents the pathways that are affected, and x-axis is -log 10(FDR), number, and % of mRNAs are represented next to each of the bar plots. Pathways that are affected by enriched mRNAs are related to platelet activation, platelet aggregation, granule content and cytoskeleton function. (**C**) Biocarta, KEGG and GO molecular pathway analysis are represented, based on the miRNAs that are absent in the LPEVs. Y-axis represents the pathways that are affected, and x-axis is -log 10(FDR), number and % of mRNAs are highlighted next to each of the bar plots. Pathways that are related to depleted mRNAs are related to genes involved in oxidative stress response, cellular respiration, and hypoxia. (**D**) Select miRNAs and related genes and pathways regulated are represented. Functional pathway analysis of the underrepresented miRNAs - miR-155, miR-199 A, and miR-200 C revealed enrichment in targets involved in NFκB signaling, angiogenesis, WNT signaling, VEGF signaling, leukocyte-endothelial transmigration, endothelial cell migration, endothelial cell proliferation, cell adhesion, PDGFβ signaling, and TGFβ signaling *[miRNA pathways representation created with BioRender.com]*

Transcriptomic content of LPEVs and platelets revealed 381 protein-coding mRNAs that were differentially represented, with 311 mRNAs enriched and 70 depleted in LPEVs relative to platelets (Fig. 5A). The enriched mRNAs in LPEVs encode proteins involved in platelet activation, cytoskeletal dynamics, and platelet aggregation, while the depleted mRNAs are associated with cellular respiration, reactive oxygen species (ROS) generation, and mitochondrial function (Fig. 5B and C). In addition, 198 microRNAs (miRNAs) were differentially present in LPEVs compared to platelets, with 33 miRNAs enriched and 165 depleted in LPEVs (Fig. 5A). Functional pathway analysis of the underrepresented miRNAs revealed their participation in processes, such as, NFκB signaling, angiogenesis, WNT signaling, VEGF signaling, leukocyte-endothelial transmigration, endothelial cell migration, cell adhesion, PDGFβ signaling, and TGFβ signaling. Among the differentially expressed miRNAs, miR-155, miR-199 A, and miR-200 C ranked highest based on the number of overlapping pathway targets. These miRNAs, along with their associated genes and affected pathways, are illustrated in Fig. 5D.

### LPEVs improve clotting function in whole blood from healthy donors and injured patients

**Fig. 6:**
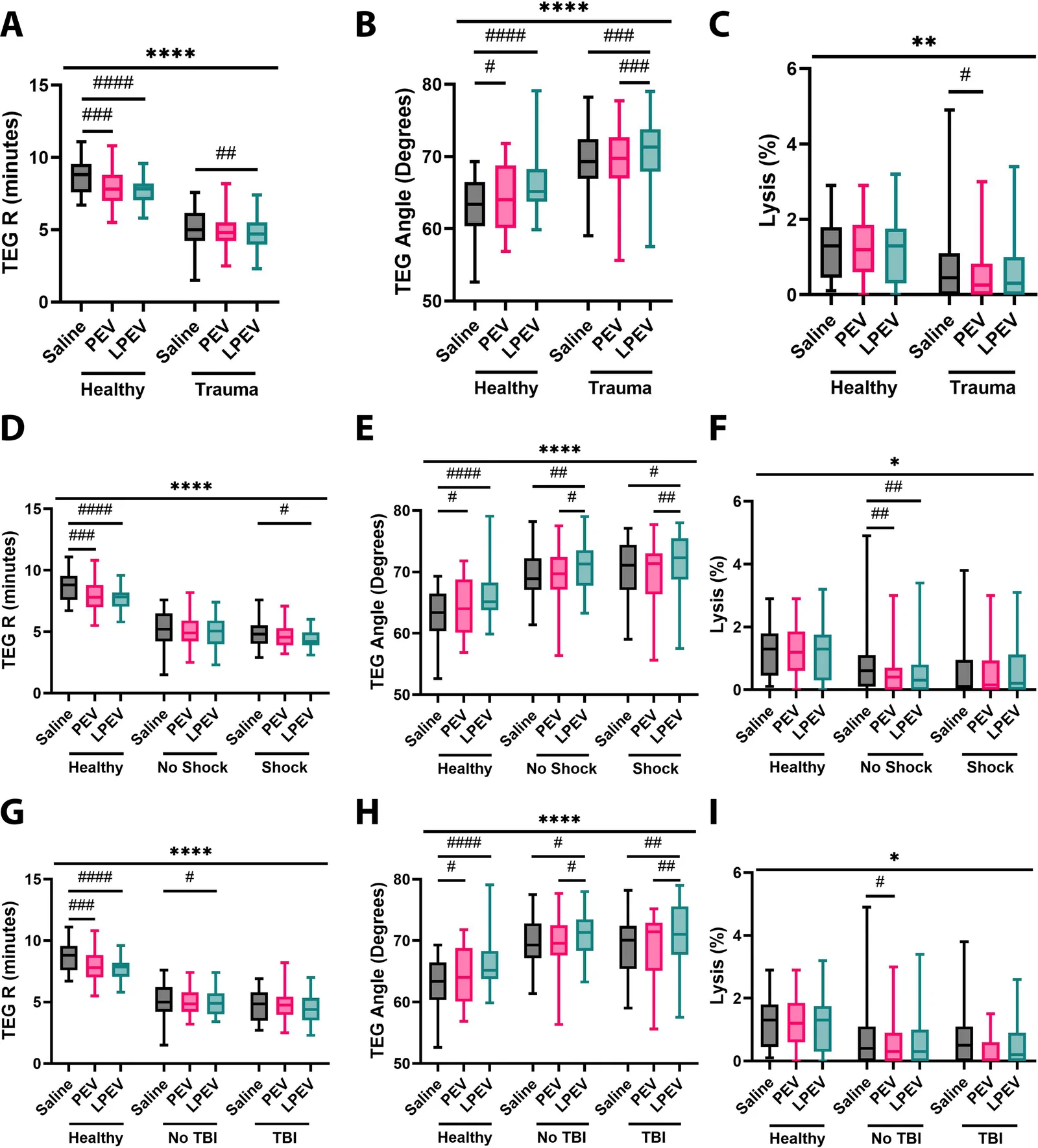
PEVs and LPEVs improve clotting function in ex vivo treated whole blood from healthy donors and injured patients. Whole blood was collected from healthy donors or injured patients and treated with saline, PEV, or LPEV (~ 10^11^ particles/mL) and subjected to TEG to assess hemostatic function. Ex vivo treatment effects in whole blood from injured patients were compared to healthy donors (**A-C**) or stratified by presence of shock (**D-F**) or TBI (**G-I**). **A**) R-time: Repeated measures mixed effect analysis (REML), significant interaction between treatment and group (healthy vs. trauma), *p* = 0.0029, F(2, 163) = 6.069; significant effect of treatment, *p* < 0.0001, F(2, 163) = 19.55; significant effect of group, *p* < 0.0001, F(1, 86) = 102.4. **B**) Angle: REML, significant interaction between treatment and group (healthy vs. trauma), *p* = 0.0368, F(2, 158) = 3.371; significant effect of treatment, *p* < 0.0001, F(2, 158) = 17.96; significant effect of group, *p* < 0.0001, F(1, 85) = 21.98. **C**) Lysis: REML, significant effect of group (healthy vs. trauma), *p* = 0.0060, F(1, 87) = 7.923. *N* = 17 healthy donors, *n* = 75 injured patients (**A-C**). **D**) R-time: REML, significant interaction between treatment and group (healthy vs. shock vs. no shock), *p* = 0.0117, F(4, 151) = 3.346; significant effect of treatment, *p* < 0.0001, F(2, 151) = 17.25; significant effect of group, *p* < 0.0001, F(1, 80) = 53.03. **E)** Angle: REML, significant effect of treatment, *p* < 0.0001, F(2, 164) = 21.46; significant effect of group, *p* < 0.0001, F(2, 88) = 11.61. **F**) Lysis: REML, significant effect of treatment, *p* = 0.039, F(1.940, 146.5) = 3.506; significant effect of group (healthy vs. shock vs. no shock), *p* = 0.0139, F(2, 81) = 4.506. *N* = 17 healthy donors, *n* = 27 shock (shock index above 1), *n* = 48 no shock (**D-F**). **G**) R-time: REML, significant interaction between treatment and group (healthy vs. TBI vs. no TBI), *p* = 0.0136, F(4, 161) = 3.245; significant effect of treatment, *p* < 0.0001, F(2, 161) = 16.38; significant effect of group (healthy vs. TBI vs. no TBI), *p* < 0.0001, F(2, 85) = 52.78. **H**) Angle: REML, significant effect of treatment, *p* < 0.0001, F(2, 156) = 20.76; significant effect of group (healthy vs. TBI vs. no TBI), *p* < 0.0001, F(2, 84) = 11.03. **I**) Lysis: REML, significant effect of treatment, *p* = 0.0133, F(1.954, 157.3) = 4.486; significant effect of group (healthy vs. TBI vs. no TBI), *p* = 0.0153, F(2, 86) = 4.390. *N* = 17 healthy donors, *n* = 22 TBI, *n* = 53 no TBI (**G-I**). All comparisons were done by repeated measures mixed effect analysis (REML), with Tukey’s multiple comparisons test as shown on the graphs * Represents between group comparisons (healthy vs. injured, effect of injury) and # represents within group comparisons (effect of treatment). */# *p* < 0.05, **/## *p* < 0.01, ### *p* < 0.001, ****/#### *p* < 0.0001

To explore the therapeutic potential of the LPEVs in bleeding patients, we sought to determine if LPEVs altered the clotting profile in human whole blood. We obtained samples from healthy donors (*n* = 17) and injured patients (*n* = 75) and treated those samples ex vivo with PEV and LPEVs. The injured patient samples came from patients that were 44±27.5 years old (median ± IQR), primarily male, who had a median (± IQR) injury severity score (ISS) of 10±20. Of these 75 patients, 22 had TBI and 27 had evidence of shock (shock index above 1) **(**Supplemental Table 1). PEV (non-lyophilized) and LPEV treatment of whole blood from both healthy donors and injured patients improved clot initiation parameters by TEG, with shorter R time and increased alpha angle (Fig. 6A and B), but without significant effects on clot lysis (LY30) (Fig. 6C). Patients were further stratified by presence of shock (Fig. 6D and F) or TBI (Fig. 6G and I), where LPEV treatment decreased R time in the whole blood from patients with shock (Figs. 6D), and both PEV and LPEV increased alpha angle regardless of stratification by shock or TBI (Fig. 6B, E and H). Treatment with PEV or LPEV of whole blood from injured patients (Fig. 6C), without shock (Fig. 6F), or TBI (Fig. 6I) had decreased clot lysis time (LY30).

### LPEVs improve aggregation of human platelets

**Fig. 7:**
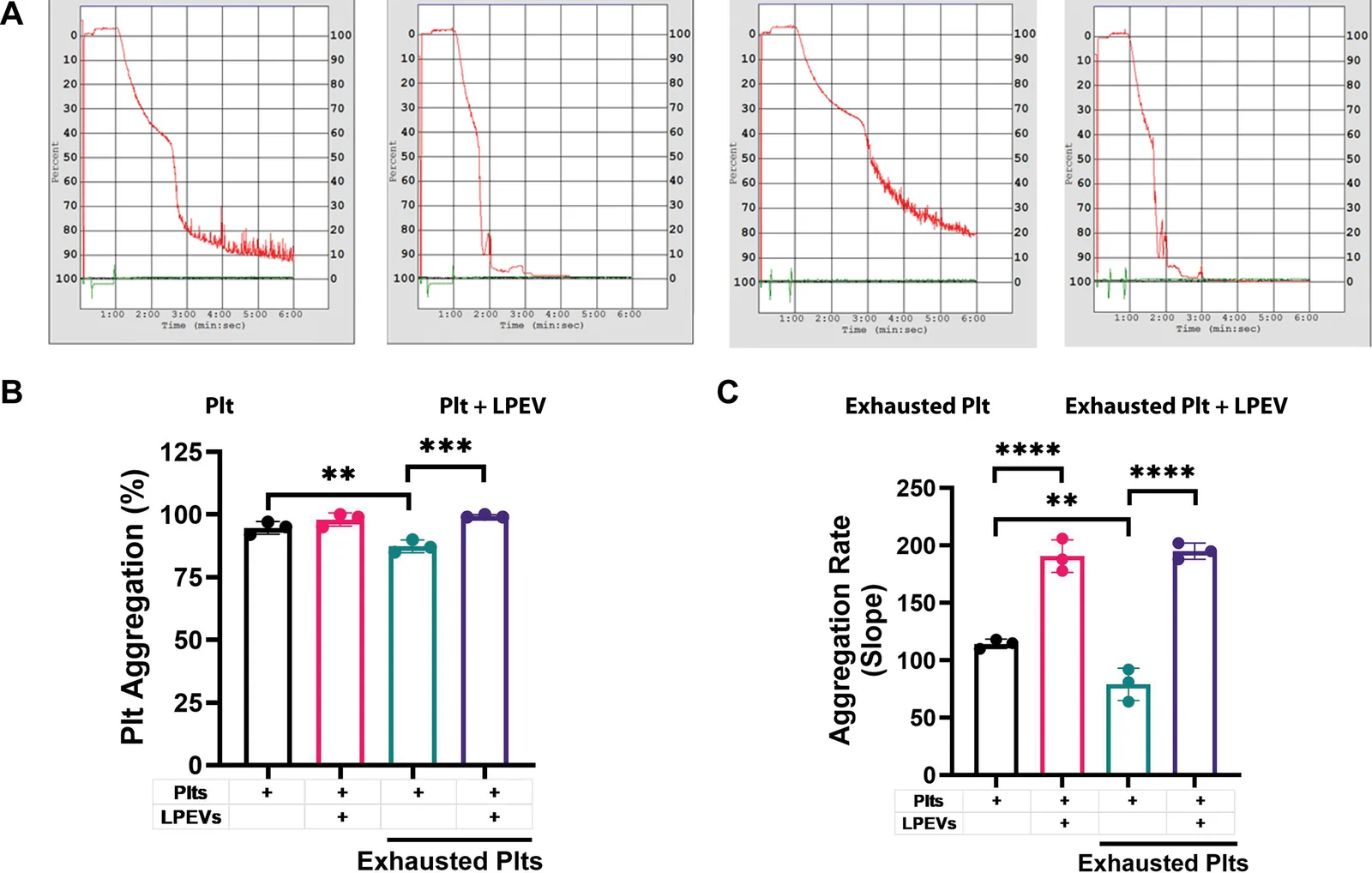
LPEVs improve aggregation of human platelets. (**A**) Tracings of light aggregometry using 2 × 10^8^ platelets or “dysfunctional platelets” treated with 3 × 10^11^ particles/mL LPEVs and thrombin as agonist. (**B**) Dysfunctional platelets aggregated less as compared to regular Plts and treatment with LPEVs increased aggregation in the dysfunctional Plts but did not affect regular Plts, RM-two-way ANOVA, significant treatment x platelet interaction *p* = 0.0052, F [1, 4] = 30.73, Sidak’s multiple comparisons test as indicated on the graph, **p = 0.0077, ***p = 0.0008. (**C**) Rate of aggregation was improved in both platelet groups when treated with LPEV. Dysfunctional platelets aggregated at a slower rate as compared to platelets. RM-two-way ANOVA, significant treatment x platelet interaction *p* = 0.0129, F [1, 8] = 10.14, Sidak’s multiple comparisons test as indicated on the graph, **p = 0.0078, ****p < 0.0001, *n* = 3 technical replicates per group. This experiment has been repeated three times with different pooled platelet donors and similar effects of LPEVs were observed

To mimic platelet dysfunction following trauma, “dysfunctional” platelets were generated by activating them with CaCl_2_. These platelets exhibited reduced aggregation capacity, and a slower aggregation rate compared to fresh apheresis platelets, similar to what has been noted in trauma patient platelets [43–45]. Notably, LPEV treatment restored both the degree and rate of aggregation in dysfunctional platelets, achieving levels comparable to those observed in fresh platelets (Fig. 7A and C).

### GPIIb/IIIa signaling is critical for supporting LPEV-mediated platelet aggregation over time but not endothelial barrier permeability

**Fig. 8:**
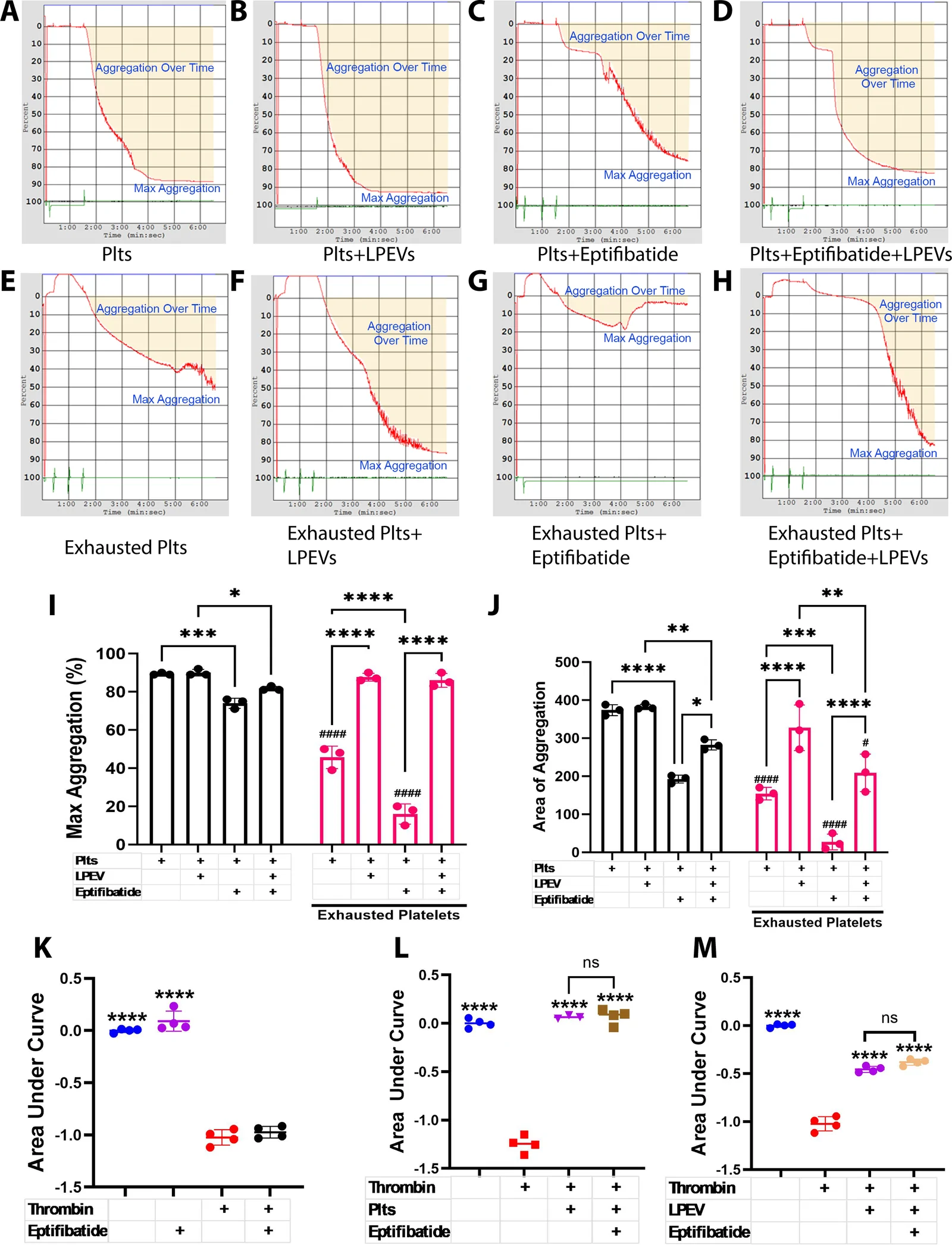
GPIIb/IIIa signaling via LPEV is critical for supporting platelet aggregation over time but not vascular stability. (**A**-**D**) Tracings of light aggregometry using 2 × 10^8^ platelets treated with 1 × 10^11^particles/mL LPEVs and/or eptifibatide with thrombin as agonist. **(E-H)** Tracings of light aggregometry using 2 × 10^8^ “dysfunctional platelets” treated with 1 × 10^11^particles/mL LPEVs and/or eptifibatide and thrombin as agonist. (**I**) Dysfunctional platelets aggregated less as compared to regular Plts. Two-way ANOVA, significant treatment x platelet interaction *p* < 0.0001, F [3, 16] = 123.1. Between group comparisons were done with Sidak’s multiple comparisons test as indicated on the graph, # vs. Plt group, ####*p* < 0.0001, Control groups, t [16] = 15.83, ####*p* < 0.0001, Eptifibatide groups t [16] = 21.02. Eptifibatide affected aggregation of both fresh and dysfunctional platelets. Within group comparisons were made with Tukey’s multiple comparisons test as indicated on the graph, Plt group: ***p = 0.0002, (*q* = 7.860, *df* = 16), dysfunctional Plt group: ****p < 0.0001, (*q* = 15.21, *df* = 16). LPEV treatment increased the percentage aggregation in dysfunctional platelets and also dysfunctional platelets treated with eptifibatide. Within group comparisons were made with Tukey’s multiple comparisons test as indicated on the graph, dysfunctional Plt group: control vs. LPEV ****p < 0.0001, (*q* = 21.53, *df* = 16), eptifibatide vs. eptifibatide + LPEV ****p < 0.0001, (*q* = 35.88, *df* = 16). Interestingly, the percentage aggregation in the normal Plts treated with eptifibatide and LPEV was significantly lower than LPEV alone. Plt group eptifibatide vs. eptifibatide + LPEV *p = 0.0367, (*q* = 4.272, *df* = 16). (**J**) Area of aggregation was significantly reduced in dysfunctional platelets as compared to regular Plts. Two-way ANOVA, significant treatment x platelet interaction *p* = 0.0006, F [3, 16] = 9.935. Between group comparisons were done with Sidak’s multiple comparisons test as indicated on the graph, # vs. Plt group, ####*p* < 0.0001, control groups, t [16] = 8.913, ####*p* < 0.0001, eptifibatide groups, t [16] = 6.706, #*p* = 0.0345, eptifibatide+LPEV groups, t [16] = 2.985. Eptifibatide decreased area aggregation of both fresh and dysfunctional platelets. Within group comparisons were made with Tukey’s multiple comparisons test as indicated on the graph, Plt group: ****p < 0.0001, (*q* = 10.41, *df* = 16), dysfunctional Plt group: ***p = 0.0005, (*q* = 7.285, *df* = 16). LPEV treatment increased the area of aggregation in platelets treated with eptifibatide. Within group comparisons were made with Tukey’s multiple comparisons test as indicated on the graph, Plt group: *p = 0.0105, (*q* = 5.159, *df* = 16). Nonetheless, this effect of LPEV was significantly lower than treatment with LPEV alone. Within group comparisons were made with Tukey’s multiple comparisons test as indicated on the graph, Plt group: **p = 0.0047, (*q* = 5.721, *df* = 16). LPEV treatment increased the area of aggregation in dysfunctional platelets and also dysfunctional platelets treated with eptifibatide. Within group comparisons were made with Tukey’s multiple comparisons test as indicated on the graph, dysfunctional Plt group: ****p < 0.0001, control vs. LPEV (*q* = 9.931, *df* = 16), ****p < 0.0001, eptifibatide vs. eptifibatide+LPEV, (*q* = 10.42, *df* = 16). Nonetheless, this effect of LPEV was significantly lower than treatment with LPEV alone. Within group comparisons were made with Tukey’s multiple comparisons test as indicated on the graph, dysfunctional Plt group: **p = 0.0010, (*q* = 6.796, *df* = 16). *n* = 3 technical replicates per group. This experiment has been repeated three times with different pooled platelet donors and similar effects of LPEVs were observed. (**K**) Eptifibatide has no effect on permeability in BECs. One-way ANOVA with Tukey’s post-hoc analysis, *p* < 0.0001, F [4, 14] = 146.9, * vs. thrombin (Thr), ****p < 0.0001. (**L**) Plts mitigated the thrombin induced barrier disruption independent of eptifibatide. One-way ANOVA with Tukey’s post-hoc analysis, *p* < 0.0001, F [3, 11] = 377.8, * vs. thrombin (Thr), ****p < 0.0001. (**M**) LPEVs mitigated the thrombin induced barrier disruption independent of eptifibatide. One-way ANOVA with Tukey’s post-hoc analysis, *p* < 0.0001, F [3, 12] = 364, * vs. thrombin (Thr), ****p < 0.0001

Consistent with our observation (Fig. 7A-B), dysfunctional platelets exhibit diminished aggregation as compared to apheresis platelets. LPEVs mitigate the aggregation related deficit in dysfunctional platelets (Fig. 8A-J, Supplemental Fig. 5A-J). One of the proteins that is conserved between the platelets and LPEVs is GPIIb/IIIa. To elucidate if GPIIb/IIIa is involved in supporting aggregation of dysfunctional platelets by LPEVs, we blocked the GPIIb/IIIa signaling pathway with eptifibatide (Fig. 8). Eptifibatide reduces aggregation of both fresh platelets and dysfunctional platelets (Fig. 8A-J). LPEVs were found to mitigate the effect of eptifibatide in dysfunctional platelets on both outcomes measured (Fig. 8I-J). Interestingly, eptifibatide suppressed the LPEVs effect on aggregation over time as compared to LPEV-treatment alone, suggesting that GPIIB/IIIA may be involved in the enhancement of dysfunctional platelet aggregation by LPEV (Fig. 8J). In contrast, blockade of GPIIb/IIIa had no effect on endothelial monolayer permeability (Fig. 8K). Furthermore, blockade of GPIIb/IIIa on fresh platelets or LPEVs did not change their protective effect on thrombin induced endothelial barrier disruption (Fig. 8L-M).

Another pathway that supports platelet aggregation is the cyclooxygenase (COX-1) pathway [46]. As seen in Fig. 8, eptifibatide delayed but did not abolish LPEV enhanced aggregation, suggesting that other pathways are involved. Thus, we inhibited the COX-1 pathway with indomethacin and observed that it only affected aggregation of dysfunctional platelets and not fresh platelets (Supplemental Fig. 5). LPEVs were found to improve aggregation and mitigate the suppressive effects of indomethacin in dysfunctional platelets on both amount of aggregation and rate of aggregation (Supplemental Fig. 5I-J). Interestingly, indomethacin did not impact the effect of LPEVs on dysfunctional platelet aggregation, suggesting the role LPEV on platelet aggregation is independent of this pathway. Furthermore, blockade of COX signaling on LPEVs did not change their protective effect on thrombin induced endothelial barrier disruption and permeability (Supplemental Fig. 5K-L).

**Fig. 9:**
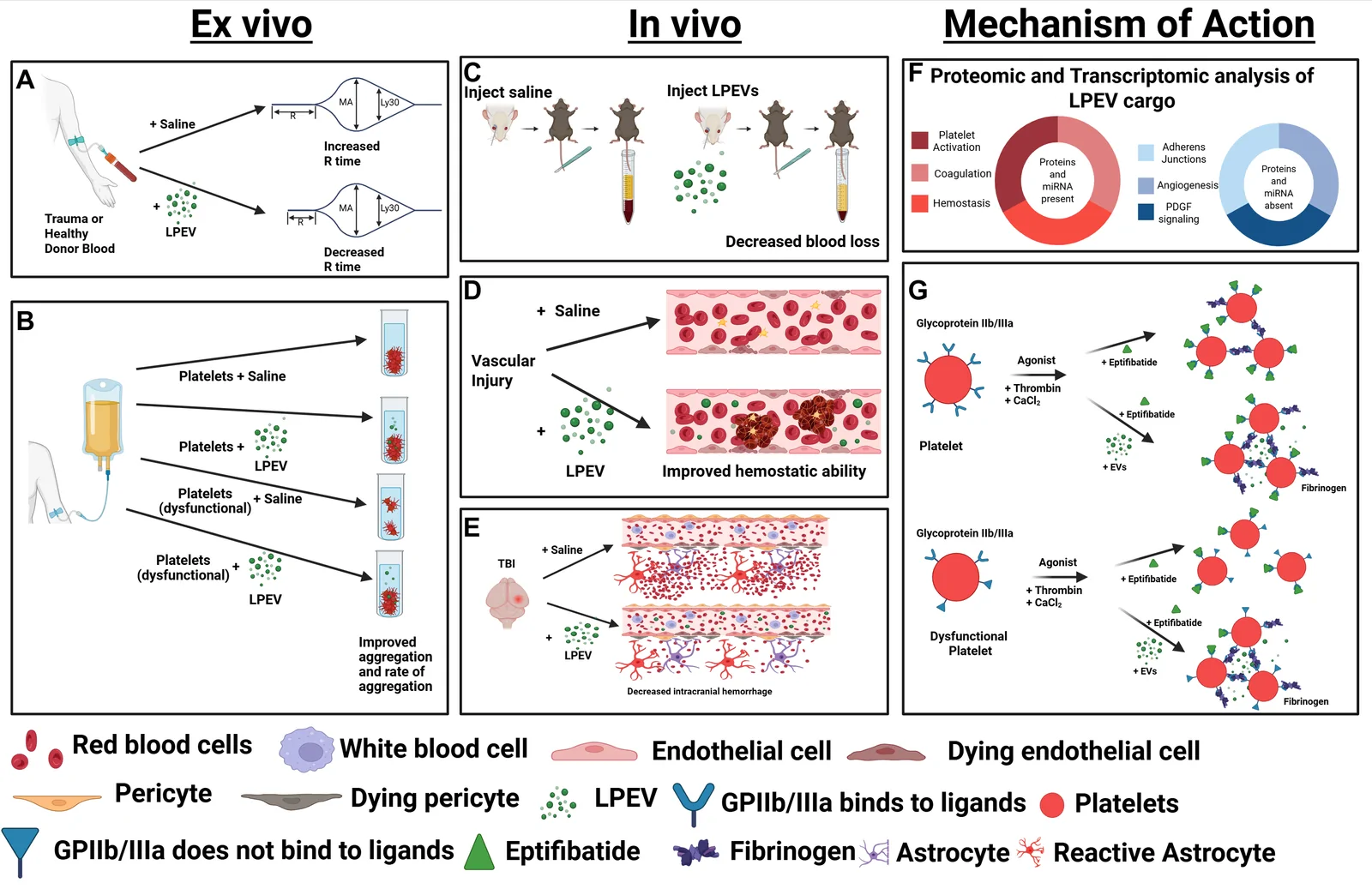
Schematic summary of hemostatic effects of LPEVs and mechanism of action. (**A**) LPEVs decreased R time in patient blood and healthy donors. (**B**) LPEVs enhance aggregation of dysfunctional platelets, surrogate marker of hemostasis. (**C**) LPEVs are hemostatic and decrease blood loss in a murine tail snip model. (**D**) Improved clot formation and time to clot in LPEV treated group in cremaster muscle vascular injury model. (**E**) Decreased ICH after TBI in LPEV treated animals. (**F**) Multiomic analysis suggests retention of cargo platelet proteins and miRNA related to coagulation, hemostasis and platelet aggregation in LPEVs. But loss of proteins and miRNA related to vascular stability. (**G**) Inhibition of GPIIb/IIIa on platelets reduces aggregation but LPEV treatment rescues this deficit. Dysfunctional platelets lose the ability to aggregate as compared to normal platelets and treatment with LPEV rescues he aggregation in dysfunctional platelets

We have summarized the findings of the hemostatic potential of LPEVs in Fig. 9. Based on the multiomics data we hypothesize that the differential cargo carried by LPEVs may explain the divergent effect seen in TBI model. Early investigation has led to GPIIb/IIIa pathway as a marker for the hemostatic potential of LPEVs.

## Discussion

In this study, we sought to explore the therapeutic potential of LPEVs in trauma related bleeding. The rationale for studying a dried EV product is based upon our previous findings demonstrating the hemostatic and therapeutic potential of the fresh EV product in trauma and the global need for logistically superior blood products that can attenuate bleeding without rapid expiration and the requirement for cold storage. Unlike conventional blood products, LPEVs have the potential to offer extended shelf life, room-temperature storage, and rapid deployment in austere, prehospital, military and civilian resource-limited settings, where access to refrigerated blood products is limited. In this paper we utilized an established pre-clinical model of TBI that allows for the evaluation of bleeding related endpoints, such as, ICH, neuroinflammation, and BBB permeability, which is related clinically to cerebral edema.

We find, in vitro, that LPEVs significantly mitigate thrombin induced intracellular gaps and permeability in brain endothelial cells, both established measures of vascular barrier integrity. In vivo, LPEVs demonstrated potent hemostatic activity in murine bleeding and injury models. Although modest, at the doses tested, LPEVs significantly decreased ICH but did not attenuate BBB permeability or neuroinflammation when transfused acutely after TBI. Consistent with these findings, LPEVs exhibited hemostatic potential in human blood from healthy individuals and trauma patients, significantly reducing the R-times and increasing alpha-angle in thromboelastography (TEG) assays. Multiomics analyses revealed that the hemostatic proteins as well as mRNA and miRNA cargo related to the hemostatic function of the platelets were retained in LPEVs, as compared to platelets, providing mechanistic support for the potent hemostatic effects observed both in vitro and in vivo of LPEVs. In contrast, several vasculo-protective factors normally present in platelets were markedly reduced or absent in LPEVs, explaining the lack of an effect on TBI-induced BBB permeability. To investigate the role of LPEVs in trauma-associated platelet dysfunction, we generated “dysfunctional” platelets, a model characterized by impaired aggregation and reduced aggregation kinetics, features that closely resemble the platelet dysfunction observed in trauma patients [43–45]. Notably, treatment with LPEVs restored both the magnitude and rate of aggregation in dysfunctional platelets. Mechanistic studies identified the conserved GPIIb/IIIa as a critical mediator of this effect.

PEVs are emerging as key bioactive agents in regenerative medicine due to their ability to carry pro-regenerative proteins and miRNAs, thus stimulating angiogenesis and tissue repair [28, 47–51]. In this study, LPEVs were derived from clinical grade apheresis platelets using previously described well-established methods [19, 20, 31] and lyophilized to increase shelf-life and accessibility, utilizing technology that has been previously employed to successfully dry platelets [29, 33, 52]. Consistent with our previous studies, LPEVs isolated by our methodology maintained the size of small EVs, ranging from 60 to 250 nm, with predominant population around 100 nm [19, 20]. The LPEVs displayed morphology markers, and protein content, that aligns with current guidelines on the definition of extracellular vesicles [30, 53]. Expression of membrane markers typical of platelets and EVs, were also observed by flow cytometry and mass spectrometry analysis [27, 54]. By mass spectrometry, we found that the LPEVs express multiple platelet glycoproteins, including GPIIb to the same extent as what is seen in the platelets.

Although the goal of this study was to explore the development of a novel LPEV product and not compare PEVs to LPEVs, we conducted some baseline studies to give us an idea of equivalence in function to ensure that lyophilization had not altered their hemostatic potential. Functionally, as seen with PEVs in our previous study [19], LPEVs demonstrated the ability to generate thrombin in a dose-dependent fashion. Consistent with the effect of PEVs on endothelial cell monolayers [20], LPEVs reduced thrombin induced BEC permeability and intercellular gap formation. In vivo, LPEVs were functionally active in supporting clot formation in cremaster muscle vascular injury model, similar to the effect of freeze-dried platelets [33]. We have previously shown in in vivo rodent and murine models, that platelets and PEVs attenuate vascular endothelial growth factor-A–induced vascular permeability and blood loss in tail snip model and uncontrolled bleeding model of hemorrhagic shock and trauma respectively [19, 20, 31, 32, 41, 42]. Here, similar to PEVs, LPEVs significantly reduced blood loss in tail snip bleeding model and demonstrated pro-hemostatic effects in TEG and platelet aggregation. Interestingly, while PEVs and LPEVs preserved endothelial barrier integrity in vitro, LPEVs failed to mitigate BBB permeability, in the murine TBI model, suggesting that the mechanisms governing endothelial barrier stabilization in vitro may not fully recapitulate the complexity of cells involved and BBB dysfunction found in vivo in TBI.

In vitro the hemostatic potential of LPEVs are demonstrated using an aggregometry assay [55]. To dissect the role of LPEVs in platelet aggregation without the confounding effects of whole blood, LTA assays were performed using aggregation agonists in the presence of either fresh apheresis platelets or platelets that were dysfunctional to simulate the dysfunctional, depleted state of circulating platelets after trauma. As expected, dysfunctional platelets formed smaller clots and clotted slowly compared to normal platelets. Importantly, LPEV addition reversed these aggregation defects in part. The LTA data supports the observed effects of LPEVs on hemostasis as found in the TEG analysis of trauma patient blood, the murine tail snip bleeding model, the cremaster vascular injury model and ICH after TBI.

In the context of neurotrauma, ROTEM^®^, TEG^®^, and TGA have been employed to evaluate hemostatic status in bleeding trauma patients [56–58]. Here we evaluate the effect of LPEVs not only in patients with severe injury at high risk for TIC, but also across a spectrum of physiologic states, including: “Sick controls” (patients who sustained trauma but did not meet criteria for severe injury), trauma patients with and without TBI, and with and without shock, and Healthy donors. The inclusion of this broader range of patient phenotypes was intentional, as our preliminary in vitro experiments and aggregation studies with normal and dysfunctional platelets demonstrated that LPEVs exert detectable effects on platelet aggregation. The persistence of LPEV-associated effects, although modest, in the presence of injury and TBI suggests that these vesicles facilitate coagulation across a broad range of clinically relevant settings. While the observed changes in TEG parameters are modest, they were consistently detectable and significant across multiple contexts (healthy donors and injured patients) [59–63]. The effects of LPEVs on TBI induced coagulopathy have yet to be determined [43, 64, 65].

To better understand the mechanisms of action of LPEVs, we investigated their molecular cargo. PEVs are known to contain growth factors, anti-inflammatory cytokines, chemokines, antioxidants, and microRNAs [47, 66]. Multiomics analyses comparing LPEVs to pooled apheresis platelets were conducted, as traditional methods such as western blotting or ELISA are limited by antibody availability and specificity. Mass spectrometry and pathway analysis of LPEV cargo revealed functional signatures consistent with the hemostatic effects observed in vivo. Proteins present in LPEVs are associated with platelet activation, coagulation cascades, prothrombin activation, hemostasis, platelet aggregation, and fibrinolysis. However, LPEVs did not reduce inflammation or prevent BBB disruption in TBI, likely due to the absence of vasculo-protective proteins that are retained in platelets [29], such as proteins regulating pathways of cell gap junctions, endothelial adherens junctions, inflammatory signaling, VEGF signaling, and WNT signaling. Similarly, miRNAs and mRNAs regulating NF-κB, WNT, TNF, VEGF, PDGF, and BDNF signaling were depleted in LPEVs compared to platelets. Whereas enriched mRNAs in LPEVs encoded proteins involved in hemostasis-related pathways, depleted mRNAs were associated with cellular respiration, hypoxia responses, and electron transport. We have recently shown that dried platelets, produced by similar technology, maintain the full spectrum of proteomic cargo and are protective effects on endothelial barrier permeability, ICH, neuroinflammation and astrocytosis after TBI, hence supporting the notion that the loss of the vasculoprotective factors in LPEVs is not secondary to lyophilization and is the reason for their lack of protection of the BBB in TBI [29].

To mechanistically understand how LPEVs modulate hemostasis, we sought to investigate the role of a critical receptor GPIIB/IIIa, as it was identified on the proteomic screen of platelets and LPEVs. GPIIB/IIIa signaling in LPEVs was a driver of the hemostatic potential independent of its effect on vascular stability, suggesting that the hemostatic and vascular protective activities of LPEVs are mediated through distinct mechanisms. Eptifibatide, an inhibitor of GPIIb/IIIa signaling muted platelet aggregation, suggesting that GPIIb/IIIa signaling was critical for normal as well as dysfunctional platelet populations. LPEVs rescued the aggregation deficit observed in eptifibatide treated platelets, but its effect was reduced compared to the platelets treated with LPEVs alone, suggesting the importance of GPIIb/IIIa expression on LPEV signaling for regulation of hemostasis. Interestingly, inhibition of the COX pathway with indomethacin suggested that this pathway was only relevant in dysfunctional platelets. Furthermore, LPEVs enhanced aggregation in dysfunctional platelets treated with indomethacin. Neither of these two pathways were found to be involved in the protective in vitro effects on endothelial permeability. These findings combined with multiomics analyses explain the effect of LPEVs on hemostasis but not on BBB permeability or neuroinflammation after TBI and is the first study to report the hemostatic potential of EVs in a TBI model.

Several limitations of this study should be acknowledged. First, we evaluated LPEVs in a single preclinical model of TBI. While this model is well established and clinically relevant for assessing ICH, BBB permeability, and neuroinflammation, validation in additional models of TBI and polytrauma will be important to confirm the generalizability of these findings. Second, donor-to-donor variability remains an important consideration for the development of LPEVs as a therapeutic product. As with other platelet-derived biologics, differences in donor characteristics and platelet composition may influence the biological activity and potency of LPEV preparations. Future studies focused on product standardization, potency assays, and characterization of lot-to-lot variability will be critical for clinical translation.

Another limitation is that our study focused primarily on acute outcomes. Although LPEVs modestly but significantly reduced ICH, we did not evaluate the effects of LPEVs on longer-term endpoints such as lesion volume, neurobehavioral recovery, cognitive function, or chronic neuroinflammation. Future investigations should include detailed dose-escalation studies, therapeutic window analyses, biodistribution studies, and assessments of thrombotic risk and immunogenicity. Here we have tested a single dose and at 40 min post-injury, a pre-hospital time-point in a clinical setting. We need to extend this to more delayed time-points of administration and also multiple doses investigated. Together, these studies will define the optimal dosing strategy, establish safety profiles, and further elucidate the therapeutic potential of LPEVs for the treatment of trauma-associated bleeding and TBI.

## Conclusion

LPEVs demonstrated potent hemostatic activity in murine bleeding and injury models and in human blood from healthy individuals and trauma patients. Although modest, at the doses tested, LPEVs significantly decreased ICH but did not attenuate BBB permeability or neuroinflammation when transfused acutely after TBI. Multiomics analyses revealed the selective cargo of hemostatic proteins retained in LPEVs, and loss of several vasculo-protective factors as compared to platelets. GPIIb/IIIa may be a critical mediator of the hemostatic effect, independent of its effect on endothelial permeability. These findings combined with multiomics analyses suggest that the hemostatic and vascular protective activities of LPEVs are mediated through distinct mechanisms. This is the first study to report the hemostatic potential of LPEVs in a TBI model.

LPEVs present multiple translational and logistical advantages as a hemostatic therapy. The ability to deliver a safe, infusible, and shelf-stable hemostatic agent like LPEVs in pre-hospital or battlefield settings could allow for earlier intervention post-TBI. Furthermore, LPEVs could serve as an adjunct to conventional blood products, improving hemostasis in bleeding patients where platelet availability is limited.

## Supplementary Information

Below is the link to the electronic supplementary material.

Supplementary Material 1

Supplementary Material 2

Supplementary Material 3

Supplementary Material 4

## Data Availability

RNA-seq data were deposited at the following GEO Database with the accession number GSE262834 (pending upon acceptance).

## Abbreviations

TBI: Traumatic brain injury
ICH: Intracranial hemorrhage
LPEV: Lyophilized platelet-derived extracellular vesicles
BBB: Blood brain barrier
PEVs: Platelet-derived extracellular vesicles
EVs: Extracellular vesicles
BECs: Brain endothelial cells
NTA: Nanoparticle tracking analysis
SEM: Scanning electron microscopy
LC-MS/MS: Liquid chromatography-tandem mass spectrometry
ECIS: Electric cell-substrate impedance sensing system
CCI: Controlled cortical impact
ED: Emergency department
TEG: Thromboeleastography
LTA: Light transmission aggregometry
Plts: Platelets
TEER: Trans endothelial electrical resistance
GFAP: Glial fibrillary acidic protein
Iba1: Ionized calcium-binding adaptor molecule 1
VEGF: Vascular endothelial growth factor
TNFα: Tumor necrosis factor alpha
ROS: Reactive oxygen species
mRNA: Messenger ribonucleic acid
miRNA: Micro ribonucleic acid
PDGFβ: Platelet derived growth factor beta
TGFβ: Transforming growth factor beta
ISS: Injury severity score
COX: Cyclooxygenase
TGA: Thrombin generation assay
ROTEM: Rotational thromboelastometry
TIC: Trauma induced coagulopathy
BDNF: Brain-derived neurotrophic factor
