# Supplementary material for: Lyophilized platelet derived extracellular vesicles promote hemostasis and attenuate intracranial hemorrhage following traumatic brain injury
Source: J Transl Med. 2026 Jul 20;24:1308. doi: 10.1186/s12967-026-08638-5 (PMC13673692; doi:10.1186/s12967-026-08638-5)
Supplement: Supplementary file 1 [file 12967_2026_8638_MOESM4_ESM.docx]

**The PDF file includes:**

Materials and Methods

Supplemental Figures 1-5

Supplemental Table 1

Legends for Movies S1 to S3

**Other Supplementary Material included are:**

Movies S1 to S3

**Supplemental Methods**

**Generation, Isolation, Purification, and Lyophilization of LPEVs**

*Apheresis Platelet Collection and PEV Generation*

Leukoreduced, irradiated apheresis platelet units (APUs) were acquired from an FDA licensed blood bank. LPEVs were isolated from clinical grade apheresis platelets that were tested for safety and infectious contamination. For each LPEV lot, six APUs from four unique donors (unknown sex) were collected in plasma and anticoagulant citrate dextrose solution A (ACD-A). Platelets from 3-5 donors were pooled for PEV isolation, to provide a more homogenous product and to prevent donor-to-donor variability.

*PEV Isolation and Lyophilization*

The extracellular vesicles (EVs) were isolated by removing platelets via centrifugation (2500RCF for 20 minutes at room temperature). The EVs were generated from non-pooled platelets, but after the EV generation steps the supernatant material was pooled together. The supernatant from the 6 APUs was pooled and filtered to remove any remaining cell debris using dead-end filtration with a 0.45-μm cellulose acetate membrane (Corning, NY). The pooled PEV isolate was then processed using tangential flow filtration (TFF). TFF was used to purify the PEVs by removing plasma and performing buffer exchange. The PEVs were processed by constant volume diafiltration using the Millipore LabScale TFF system with a Biomax 500 kDa Pellicon polyethersulfone filter (Millipore, Billerica, MA), to remove any particles smaller than 5nm. The TFF processing conditions were a target feed pressure of 20PSI and retentate pressure of 10PSI. Three diavolumes of proprietary buffer were used to facilitate the purification and to formulate the PEVs in a solution suitable for lyophilization and long-term storage. After TFF processing, the purified PEV material was 0.2-μm sterile filtered and then 1mL aliquots were aseptically filled into 5mL glass vials. The glass vials were then lyophilized to produce a freeze-dried product with less than 2% residual moisture and stoppered under vacuum.

**Flow cytometry and Nanoparticle tracking analysis (NTA)**

LPEVs were resuspended in 1ml of particle-free water to a concentration of 1.8x10^12^ particles/mL. The LPEVs were then diluted with FACS buffer (0.1mM filtered dPBS) to a concentration of 2-8x10^9^ particles/mL. Antibodies to CD9-APC (catalog # 312107), CD41-BV510 (catalog # 303735), IgG-APC (catalog # 400121), and IgG-BV510 (catalog #400171) (BioLegend, San Diego, CA) were filtered by ultracentrifugation. Isotype controls were used to determine the level of background fluorescence. LPEVs were blocked with mouse gamma globulin (1mg/mL, Invitrogen) for 10min at RT. After blocking, LPEVs were incubated with the above antibodies or Annexin-BV605 stain (catalog #563974) (BioLegend) at 1:100 in a volume of 100ul. After 60 minutes of staining at 22°C, LPEVs were filtered through qEV 70nM columns to remove unbound antibodies (Izon, Medford, MA). Samples were analyzed on a Cytek Aurora benchtop flow cytometer equipped with 355 nm, 405 nm, 488 nm, 561 nm, and 640 nm excitation lasers (Cytek, Fremont, CA). Samples were acquired to count 2x10^6^ events at a 10,000 events/second flow rate. For the size confirmation of LPEVs, Apogee Silica reference beads, ranging in size from 80 nm to 1300 nm were used (Apogee, Catalogne, Spain). The voltages for FSC (426) and SSC (3003) gating was optimized for this range of bead sizes. Data were analyzed using FlowJo software (Tree Star, Inc., Ashland, OR).

**Nanoparticle tracking analysis (NTA)**

The concentration and size distribution of LPEVs were evaluated using a NanoSight NS300 instrument (Malvern). The samples were diluted in 1:1000 in dPBS. Five videos were recorded at camera level 11, acquisition time 25 FPS, and detection threshold of 6. Data analysis was performed with NTA software.

**Thrombin Generation Assay (TGA)**

Thrombin generation was measured in triplicate using the Calibrated Automated Thrombogram (CAT) method with a 96-well plate. Each well received 40 μL of sample, 40 μL of 60% Octaplas (Octapharma, NDC# 68982-955-01) in phosphate-buffered saline (PBS, Corning, 21-040-CV), and 20 μL of Stago PRP-reagent (tissue factor with no phospholipids, #86196). Octaplas is a solvent/detergent treated plasma with no phospholipids and was added as a source of coagulation factors. To initiate the coagulation cascade, injection of 20 μL of fluorogenic substrate in calcium buffer (Stago FluCa-reagent, #86197) was performed by the instrument immediately prior to measurement initiation. This was the only assay in which Octaplas was added to the LPEVs as a source of coagulation factors. For all other assays, only reconstituted LPEVs were used.

PEV and LPEV-depleted supernatant samples were tested on TGA. The samples were plated for TGA as undiluted samples or diluted samples. Diluted samples were diluted 1:5, 1:205, or 1:300 in PBS prior to being plated for TGA. PEV and LPEV-depleted supernatant samples were prepared via ultracentrifugation at 100,000 RCF for 1 hour at 4°C (supernatant collected, pellet discarded). A negative control sample of 40 μL of 60% Octaplas was also tested on TGA (80 μL total of 60% Octaplas in the well).

**Platelet and LPEV content analysis**

All preparation and LC-MS/MS runs were performed by System Biosciences (SBI, Palo Alto, CA).

*Proteomic analysis of Platelets and LPEVs:*

In-gel digestion

Samples (lysates) were run on SDS-PAGE as gel plugs. Each gel plug was subjected to reduction with 10mM DTT for 30 min at 60℃, alkylation with 20mM iodoacetamide for 45min at room temperature in the dark and digestion with trypsin (sequencing grade, Thermo Fisher Scientific, Waltham, MA), and incubation over-night at 37°C. Peptides are extracted twice with 5% formic acid, 60% acetonitrile and dried under vacuum.

Liquid chromatography-tandem mass spectrometry (LC-MS/MS)

Samples are analyzed by nano LC-MS/MS using an Eclipse tribrid mass spectrometer interfaced with a Ultimate 3000 RSLCnano chromatography system (Thermo Fisher Scientific).  Samples were loaded on to a fused silica trap column (Acclaim PepMap 100, 75umx2cm, Thermo Fisher Scientific). After washing for 5 min at 5 µl/min with 0.1% TFA, the trap column was brought in-line with an analytical column (Nanoease MZ peptide BEH C18, 130A, 1.7um, 75umx250mm, Waters) for LC-MS/MS.  Peptides were fractionated at 300 nL/min using a segmented linear gradient 4-15% B in 30min (A: 0.2% formic acid; B: 0.16% formic acid, 80% acetonitrile), 15-25%B in 40min, 25-50%B in 44min, and 50-90%B in 11min.  Mass spectrometry data was acquired using a data-dependent acquisition procedure with a cyclic series of a full scan from 350-1500 with resolution of 120,000 control (AGC) target 5E4, maximum injection time 100 ms. The top S (3 sec) duty cycle and dynamic exclusion of 30sec were used for selection of Parent ions for MSMS in the HCD cell with, relative collision energy 30% and scanned in the orbitrap with resolution of 30,000. Nano LC-MSMS Raw files were analyzed in Proteome discoverer (v.3.0) using Sequest HT search engine against uniprot human reference proteome database (83,607 entries) as well as a database composed of common lab contaminants (199 entries, https://www.thegpm.org/crap/).  Digestion protease was set as trypsin full digestion with 1 misscut allowed. Precursor mass tolerance was set at 10ppm and fragment mass tolerance were set at 0.02 Dalton. Carboxyiodomethyl on cysteine was set as static modification, acetylation, methionine loss and acetylation plus methionine loss were set as dynamic modifications at the protein terminus. Oxidation on methionine was set as dynamic modification. Percolator was used to validate results with strict target false discovery rate set to 0.01 and relaxed target rate set to 0.05 for both peptides and proteins. Peptides were grouped into protein groups using strict parsimony principle. Summed Intensity of all belonging unique and razor peptides were used to represent protein abundance. Protein abundances were normalized to total peptides amount of that sample and reported as “normalized abundance”. No scaling was applied.

*Transcriptomic analysis of Platelets and LPEVs:*

All isolations and analyses were performed by System Biosciences (SBI, Palo Alto, CA).

RNA isolation

Day 1 human apheresis platelets and LPEVs were lysed and crude RNA extracted using SBI’s spin column-based RNA Purification Kit. RNA quality and quantity were checked on Bioanalyzer (Agilent, Santa Clara, CA) using the RNA Pico kit and assessed for presence of small RNA.

Library generation and sequencing

Total RNA population, starting with as little as 1 ng of total RNA, was entered into small RNA library prep using Qiagen small RNA library prep kit. Successful libraries were purified by gel extraction to ensure the correct size was isolated. Libraries were amplified by qPCR and assessed for viability by the concentration (>1 nM) to be moved on to sequencing. Next Gen Sequencing was performed on Illumina NextSeq with SE75 (Single-end, 75 bp) at an approximate depth of 10-15 million reads per sample.

**Quantitation of endothelial barrier permeability in monolayer cell culture**

Human brain microvascular endothelial cells (BECs) (Angio-Proteomie, Boston, MA) were expanded and maintained using Endothelial Growth Medium (Angio-Proteomie). The integrity of BEC monolayers was measured using an electric cell-substrate impedance sensing system (ECIS 1600, Applied BioPhysics, Troy, NY). An increase or decline in transendothelial electrical resistance (TEER) across the cell monolayers indicated decreased or increased endothelial paracellular permeability. BECs were seeded at a density of 50,000 cells//well and grown to confluence (2-3 days) on L-cysteine reduced, 96-well plates containing electrodes in each well. LPEVs were reconstituted with 1 ml ultrapure sterile water and used within one hour of reconstitution. Leukoreduced apheresis Plts stored in plasma were obtained from Vitalant (Denver, CO). Plts were washed with PBS and activated with 1mM CaCl_2_ before treatment. In an incubator set to maintain 37 °C with 5% CO_2_, cells were treated with LPEVs (10^9^ to 10^11^ particles/ml), Plts (50x10^6^/ml), eptifibatide (Bachem, Torrance, CA), or indomethacin (Sigma, St. Louis, MO) and were challenged after 30 minutes with thrombin (2.0U/ml). For eptifibatide conditions, cells were either treated with eptifibatide (3µM) for 30 minutes prior to LPEV treatment, or LPEVs/PLTs were incubated with eptifibatide (9µM) for 30 minutes before addition to the cell monolayer. For indomethacin conditions, cells were either treated with indomethacin (20µM) for 30 minutes prior to LPEV treatment or LPEVs were incubated with 20µM indomethacin for 30 minutes before addition to the cell monolayer. Monolayer resistance at 4/16/64 kHz was analyzed in 8-min intervals for a duration of 3-4 hours, after the effect of thrombin had plateaued. A minimum of three biological replicates was conducted. Data were normalized to the mean resistance of cell monolayers measured before the treatments.

**Light transmission aggregometry**

Day 1 leukoreduced platelets (Plts) (Vitalant, Denver, CO) from 3 donors were pooled, centrifuged at 800g for eight minutes and resuspended in a fixed volume of PBS. The dysfunctional Plt condition – representing spent platelets seen *in vivo*, was generated by a 30-minute incubation of Plts with 1mM CaCl_2_ at 37°C prior to incubation with LPEV. Pooled Plts (2x10^8^) were treated with 10^11^ LPEVs/mL for 30 minutes at 37°C. All Plt conditions were then primed with 1mM CaCl_2_ for 0.5 minutes before stimulation with the agonist thrombin (0.1U/ml). Aggregation was analyzed on a Model 700 Whole Blood/Optical Lumi-Aggregometer (Chrono-log, Havertown, PA) by measuring optical density for 5 minutes and quantified as a percentage aggregation as compared to that of the vehicle control (PBS). The rate of aggregation was quantified by measuring the slope of the curve generated.

To dissect the roles of the GPIIb/IIIa pathway or cyclooxygenase pathway (COX) in LPEV induced aggregation, the effect of eptifibatide and indomethacin respectively on Plt-LPEV interaction was examined. Prior to aggregation analysis, Plts-eptifibatide conditions were generated by treating Plts with eptifibatide (9µM) for 5 minutes. Plts-indomethacin conditions were generated by treating Plts with indomethacin (20µM) for 5 minutes. To evaluate this pathway, complete exhaustion of the Plts was achieved by incubation with 10mM CaCl_2_ for 0.5 minutes, followed by treatment with 10^11^ LPEVs/mL for 0.5 minutes (according to condition), before stimulation with the agonist thrombin (0.1U/mL). Aggregation was analyzed as described above. Aggregation over time was measured as a function of aggregation time and depth. All conditions were run in triplicate, and experiment was repeated 3 separate times with 3-5 pooled donor platelets.

**Intravital microscopy of mouse cremaster vasculature injury model**

Adult male C57BL6 mice (8–12-week-old) (Jackson Laboratories, Bar Harbor, ME) were used for this study. Mice were anesthetized with an intraperitoneal injection of ketamine (90mg/kg) and xylazine (10mg/kg) and placed on temperature-controlled heating pad. The trachea was intubated using polyethylene 90 tubing (Becton Dickinson, Sparks, MD) to allow spontaneous respiration. The left femoral artery was cannulated using Instech mouse femoral cannulas (Instech Laboratories, Plymouth Meeting, PA), for the administration of anesthetic, FITC-dextran. The cremaster muscle was exteriorized, pinned to the stage and superfused with warm bicarbonate-buffered saline (131.9mM NaCl, 18mM NaHCO_3_, 4.7mM KCl, 2.0mM CaCl_2_•2H_2_O, and 1.2mM MgCl_2_) equilibrated with 5% CO_2_ in N_2_. Using a Leica DM6 FS epifluorescence microscope (Leica, Feasterville, PA) with Hamamatsu camera (Hamamatsu, Bridgewater, NJ) a post capillary venule was located for injury and analysis. After baseline imaging of a vessel (uninjured), a 50-μL bolus of 3 mg/mL FITC-tagged 70-kD dextran in saline was administered via the cannula. LPEVs or saline were administered as treatments. The vessel was injured by exposure to light, which in the presence of dextran, induces vascular injury. Vessels were imaged for up to 30 minutes. Once clot formation was complete, the time to clot (obstruct blood flow) was recorded and imaged with TRITC illumination revealing the clot. Images were captured using MetaMorph (Molecular Devices, San Jose, CA).

**Tail snip assay**

The tail snip bleeding model was performed in 8- to 10-week-old male mice (Jackson Laboratories). Isoflurane-anesthetized mice (2%) were placed on a warming pad and body temperature maintained at 37^o^C. They injected retroorbitally with vehicle (sterile saline), or LPEVs (4x10^8^ to 2x10^10^ particles/g body weight), 5 minutes prior to resection of the tail. After 5 minutes, the distal portion of the tail (~5 mm segment) was amputated and immediately immersed in 14 mL of 37°C PBS. This blood was collected for 10 minutes of bleeding time. Mice were randomized to receive the treatment, and the surgeon was blinded to the treatment delivered. Total time of bleeding was noted for each of the animals. At the end of the bleeding time, 100ul of the collected blood aliquot was measured along with a standard curve of blood volume, using the SoftMax Pro 5 Microplate Reader at 550 nm. Blood loss was quantified based on the absorbance of the sample against standard curve and corrected per g body weight of the mouse.

**Traumatic brain injury (TBI) model**

The controlled cortical impact (CCI) injury was performed on 10-12 week old C57Bl/6J male mice (Jackson Laboratories). Mice were randomized to receive injury and treatment. Surgeon was blinded to the treatment groups. Craniotomy (5mm) was performed on isoflurane-anesthetized (2%) mice and a moderate level of cortical contusion injury was performed using stereotactic device, Impactor 1 (Leica Biosystems, Buffalo Grove, IL). A 3mm piston impacted the cortex at a velocity of 4.5 m/s, depth of 1.2 mm, and dwell time of 300 ms. After injury, the impactor tip was retracted, the meninges remain intact. Control, sham operated animals were subjected to only anesthesia and surgical incision without craniotomy and impact injury. Animals were injected retroorbitally with saline or LPEVs (2x10^9^ to 2x10^10^ particles/g body weight), at 40 minutes post-injury. After the injury or sham surgery, the incision was closed and the animal was allowed to recover from anesthesia by placing it on a heating pad to maintain body temperature. At the end of surgery, the mice received 1ml of saline sub-cutaneously for hydration purposes.

**Measurement of BBB permeability and immunohistopathology**

Animals were sacrificed at 3 days post-injury to measure BBB permeability. One hour before sacrifice, animals were retro-orbitally injected with IR680 tagged 10 kD dextran (200ul, 0.5mg/mL; Thermo Fisher Scientific, Waltham, MA) and dye was allowed to circulate for 60 minutes. Mice were transcardially perfused with ice-cold PBS (30mL), brains excised and 2mm coronal sections were sliced and scanned on Odyssey CLX scanner (Licor, Lincoln, NE). Dye intensity was quantitated by dividing the slice into four quadrants (ipsilateral dorsal, ipsilateral ventral, contralateral dorsal and contralateral ventral) using the Odyssey imaging system (Licor). Total IR dye per unit area was quantitated and compared between groups and regions.

Brain slices were fixed with 4% Paraformaldehyde (PFA) overnight and dehydrated for 3-5 days in 30% sucrose solution then embedded in Optimal cutting temperature (OCT) freezing medium. Embedded brains were further sectioned to 20 µm thickness using a Leica 1950 cryostat (Leica Biosystems, Wetzlar, Germany) and sections were collected on Superfrost slides (Thermo Fisher Scientific). PFA fixed brain sections were stained with antibodies against CD68 (Bio-Rad, Hercules, CA), GFAP (Millipore, Burlington, MA), Iba-1 (Novus Biologicals, Littleton, CO) and Ter119 (RBC marker for hemorrhage, BD Pharmingen, Milpitas, CA). TRITC, Alexa Fluor 488 or Alexa Fluor 568 conjugated secondary antibodies (Thermo Fisher Scientific) were used for detection. Images were captured using a Nikon Eclipse 80i microscope (Nikon, Melville, NY) with RT-scmos camera (SPOT Imaging, Sterling Heights, MI) at 10x magnification using same exposure settings across all animals for each of the individual markers. Two slides per animal, with three sections per slide and 2-7 images per section were analyzed. For Ter119 analysis, each section was given a score as follows: score 0 = no hemorrhage, score 1 = up to 10% of the injured cortex shows bleeding, score 2 = 10-25% of the injured cortex shows bleeding, score 3 = 25-50% of the injured cortex shows bleeding, score 4 = more than 50% of the injured cortex shows bleeding. These scores were totaled per animal. For all the other markers, fluorescent intensity measurements were performed. Integrated fluorescence intensity was measured using ImageJ software and MaxEntropy method of thresholding. Percentage integrated fluorescence density was calculated as (Threshold fluorescence intensity/Total fluorescence intensity) x 100.

**Supplemental data**

**supplemental Figure 1. Scanning electron microscopy analysis.** Multiple fields of view confirm the presence of particles that are 100nm in size.

**supplemental Figure 2. Flow cytometry analysis of the LPEVs. (A)** Sizing beads were analyzed and plotted to show the different sizes. **(B)** Buffer alone control gate shows very low content of EVs. **(C)** EVs were gated as depicted in panel B, to measure all particles in the less than 240nm size range. **(D-F)** Flow cytometry analysis using control IgGs show negligible signal in quadrant 2 (Q2). **(G-I)** Flow cytometry analysis of the LPEVs showed that they expressed CD9, Annexin V, and CD41.

**supplemental Figure 3. LPEVs do not affect injury induced infiltrating or resident immune cell response at 3 days post-injury. (A)** Infiltrating macrophages were increased at the injury site, but unchanged in response to LPEV treatment. One way ANOVA, p=0.0006, F(3,22)=8.471. Left shows representative images from each group and right shows the quantitative analysis. N=6-8 per group. Activation of microglia as measured by Iba1 intensity was increased upon injury in the **(B)** penumbral region, Kruskal-Wallis test, p=0.0018, n=6-8 per group, and **(C)** hippocampus, One way ANOVA, p=0.0020, F(3,19)=7.251 with no differences between the injured-treated groups, n=5-6 per group. Left shows representative images from each group and right shows the quantitative analysis. High magnification insets shown in the upper right corner. Blue: dapi, Red: Iba1, LD: low dose LPEV, HD: high dose LPEV; columns represent mean±SD, dotted horizontal line represents sham mean and solid horizontal line represents ±SD.

**supplemental Figure 4. LPEVs do not affect injury induced astrogliosis at 3 days post-injury.** Activation of astrocytes as measured by GFAP intensity was increased upon injury but unchanged in response to LPEV treatment as compared to saline treated mice in the **(A)** penumbral region, One way ANOVA, p<0.0001, F(3,22)=29.14, and **(B)** hippocampal region, One way ANOVA, p=0.0006, F(3,22)=8.652. **(C)** No differences in GFAP expression were observed in the white matter, One way ANOVA, p=0.0536, F(3,22)=2.977. columns represent mean±SD, dotted horizontal line represents sham mean and solid horizontal line represents ±SD, n=5-8 per group. Blue in B and C – Dapi, Red – GFAP. Left shows representative images from each group in the individual area and right shows the corresponding quantitative analysis.

**supplemental Figure 5. LPEVs mitigate indomethacin induced decrease in dysfunctional platelet aggregation. (A-D)** Tracings of light aggregometry using 2x10^8^ platelets treated with 3x10^10^/mL EVs and/or endomethacin and thrombin as agonist. **(E-H)** Tracings of light aggregometry using 2x10^8^ “dysfunctional platelets” treated with 3x10^10^/mL EVs and/or endomethacin and thrombin as agonist. **(I)** Dysfunctional platelets aggregated less as compared to regular Plts. Two-way ANOVA, significant treatment x platelet interaction p<0.0001, F (3, 16) = 23.37. Between group comparisons were done with Sidak’s multiple comparisons test as indicated on the graph, # vs Plt group, #p=0.0199, Control groups, t(16) = 3.215, ####p<0.0001, indomethacin groups t(16) = 10.17. Indomethacin affected aggregation only dysfunctional platelets. Within group comparisons were made with Tukey’s multiple comparisons test as indicated on the graph, control vs indomethacin ****p<0.0001, (*q* =10.08, *df* = 16). Treatment with LPEV improved aggregation in dysfunctional platelets and dysfunctional platelets treated with indomethacin, to levels observed with normal platelets. Within group comparisons were made with Tukey’s multiple comparisons test as indicated on the graph, dysfunctional Plt group: control vs LPEV, *p=0.0287, (*q* =4.449, *df* = 16), LPEV vs indomethacin, ****p<0.0001, (*q* = 14,53, *df* = 16). Interestingly, indomethacin had no effect on LPEV treatment, LPEV vs indomethacin + LPEV, p>0.05. **(J)** Area of aggregation was significantly reduced in dysfunctional platelets as compared to regular Plts. Two-way ANOVA, significant treatment x platelet interaction p=0.0003, F(3,16) = 11.29. Between group comparisons were done with Sidak’s multiple comparisons test as indicated on the graph, # vs Plt group, ####p<0.0001, control groups, t(16) = 7.198, ##p=0.0063, LPEV groups, t(16) = 3.801, ####p<0.0001, indomethacin groups, t(16) = 10.39. #p=0.027, indomethacin+LPEV groups, t(16) = 3.104. Indomethacin decreased area aggregation only in dysfunctional platelets. Within group comparisons were made with Tukey’s multiple comparisons test as indicated on the graph, dysfunctional Plt group: *p = 0.0119, (*q* = 5.071, *df* = 16). LPEV treatment increased the area of aggregation in dysfunctional platelets and dysfunctional platelets treated with indomethacin. Within group comparisons were made with Tukey’s multiple comparisons test as indicated on the graph, dysfunctional Plt group: *p=0.0139, (*q* = 4.963, *df* = 16), ****p<0.0001, (*q* = 10.7, *df* = 16), **p=0.0047, (*q* = 10.7, *df* = 16). Interestingly, indomethacin had no effect on LPEV treatment, LPEV vs indomethacin + LPEV, p>0.05, also the increase in area of aggregation was maintained in the presence of indomethacin, **p=0.0053, (*q* = 5.629, *df* = 16). **(K)** TEER tracings of BEC monolayers treated with LPEVs or indomethacin+LPEVs, followed by thrombin challenge. **(L)** Area under the curve (AUC) quantification representing the TEER tracings of the endothelial monolayer under different treatment conditions. LPEVs mitigated the thrombin induced barrier disruption independent of indomethacin. One-way ANOVA with Tukey’s post-hoc analysis, p<0.0001, F(3, 11)=83.55, * vs thrombin (Thr), ****p<0.0001.

**Supplemental Table 1.** Data are expressed as n/total patients with data for a given variable. Percentages are expressed out of total patients in study. Continuous variables are expressed as median ± interquartile range.

| **Characteristic** |  | **Trauma** |
| --- | --- | --- |
| Age (years) |  | 44±27.5 |
| Total (n (%)) |  | 75/75 (100%) |
| Gender | Female (n (%)) | 17/75 (22.7%) |
|  | Male (n (%)) | 56/75 (74.7%) |
|  | Other/Unknown (n (%)) | 2/75 (2.6%) |
| Race | White (n (%)) | 26/75 (34.7%) |
|  | Black (n (%)) | 17/75 (22.7%) |
|  | Hispanic (n (%)) | 7/75 (9.3%) |
|  | Asian (n (%)) | 11/75 (14.7%) |
|  | Native American (n (%)) | 1/75 (1.3%) |
|  | Pacific Islander (n (%)) | - |
|  | Other (n (%)) | 12/75 (16%) |
|  | Unknown (n (%)) | 1/75 (1.3%) |
| Mechanism of Injury | Blunt (n (%)) | 41/75 (54.7%) |
|  | Penetrating (n (%)) | 34/75 (45.3%) |
| Traumatic Brain Injury (n) |  | 22/75 (29.3%) |
| Injury Severity Score |  | 9.5±20.25 |
| Admit Glascow Coma Scale |  | 14±4 |
| Body Mass Index (kg/m^2^) |  | 25.7±5.2 |
| Admit Heart Rate (beat per minute) |  | 93±31 |
| Admit SBP (mm Hg) |  | 136±30.2 |
| Shock Index |  | 0.9±0.4 |
|  | SI<1 (n) | 43/70 (57.3%) |
|  | SI >1 (n) | 27/70 (36%) |
| Plt (x10^9^/L) |  | 263±98 |
| RBC (x10^12^/L) |  | 4.5±1.1 |
| WBC (x10^9^/L) |  | 9.8±5.6 |
| Hemoglobin (g/dL) |  | 13.6±3.1 |
| INR |  | 1.1±0.1 |
| Base excess (mEq/L) |  | -1.5±5.6 |
| Transfused within 24 hrs | No (n(%)) | 59/74 (78.7%) |
|  | Yes (n(%)) | 15/74 (20%) |
| Mortality at 24 hours | Alive (n (%)) | 65/67 (86.7%) |
|  | Deceased (n (%)) | 2/67 (2.7%) |
| Mortality at 28 days | Alive (n (%)) | 42/48 (56%) |
|  | Deceased (n (%)) | 6/48 (8%) |
| Vital status at discharge | Alive (n (%)) | 45/52 (62.5%) |
|  | Deceased (n (%)) | 7/52 (9.3%) |
| Hospital days |  | 8±15 |
| Intensive Care Unit days |  | 2±7 |

**Movie 1: Untreated BEC monolayers maintain the cell density.** Representative time-lapse imaging video of untreated BEC monolayer that was assessed for gap area.

**Movie 2: BEC monolayers treated with thrombin, show loss of cell density.** Representative time-lapse imaging video of BEC monolayer treated with thrombin and assessed for gap area.

**Movie 3: BEC monolayers treated with LPEV, mitigate thrombin induced loss of cell density.** Representative time-lapse imaging video of BEC monolayer treated with thrombin and LPEV and assessed for gap area.
